# Decreased left heart flow in fetal lambs causes left heart hypoplasia and pro-fibrotic tissue remodeling

**DOI:** 10.1038/s42003-023-05132-2

**Published:** 2023-07-22

**Authors:** Miriam S. Reuter, Dustin J. Sokolowski, J. Javier Diaz-Mejia, Johannes Keunen, Barbra de Vrijer, Cadia Chan, Liangxi Wang, Greg Ryan, David A. Chiasson, Troy Ketela, Stephen W. Scherer, Michael D. Wilson, Edgar Jaeggi, Rajiv R. Chaturvedi

**Affiliations:** 1 CGEn, The Hospital for Sick Children, Toronto, ON Canada; 2 The Centre for Applied Genomics, The Hospital for Sick Children, Toronto, ON Canada; 3 Genetics and Genome Biology, SickKids Research Institute, Toronto, ON Canada; 4 Department of Molecular Genetics, University of Toronto, Toronto, ON Canada; 5 Princess Margaret Cancer Centre, University Health Network, Toronto, ON Canada; 6 Ontario Fetal Centre, Department of Obstetrics & Gynaecology, Mount Sinai Hospital, Toronto, ON Canada; 7 Department of Obstetrics & Gynaecology, University of Toronto, Toronto, ON Canada; 8 Department of Obstetrics & Gynaecology, Western University, London, ON Canada; 9 Children’s Health Research Institute, London, ON Canada; 10 London Health Sciences Centre, Victoria Hospital, London, ON Canada; 11 Department of Paediatric Laboratory Medicine, The Hospital for Sick Children, Toronto, ON Canada; 12 Department of Laboratory Medicine & Pathobiology, University of Toronto, Toronto, ON Canada; 13 McLaughlin Centre, University of Toronto, Toronto, ON Canada; 14 Labatt Family Heart Centre, Division of Cardiology, The Hospital for Sick Children, Toronto, ON Canada; 15 Department of Paediatrics, University of Toronto, Toronto, ON Canada

**Keywords:** Heart development, Congenital heart defects

## Abstract

Low blood flow through the fetal left heart is often conjectured as an etiology for hypoplastic left heart syndrome (HLHS). To investigate if a decrease in left heart flow results in growth failure, we generate left ventricular inflow obstruction (LVIO) in mid-gestation fetal lambs by implanting coils in their left atrium using an ultrasound-guided percutaneous technique. Significant LVIO recapitulates important clinical features of HLHS: decreased antegrade aortic valve flow, compensatory retrograde perfusion of the brain and ascending aorta (AAo) from the arterial duct, severe left heart hypoplasia, a non-apex forming LV, and a thickened endocardial layer. The hypoplastic AAo have miRNA-gene pairs annotating to cell proliferation that are inversely differentially expressed by bulk RNA-seq. Single-nucleus RNA-seq of the hypoplastic LV myocardium shows an increase in fibroblasts with a reciprocal decrease in cardiomyocyte nuclei proportions. Fibroblasts, cardiomyocytes and endothelial cells from hypoplastic myocardium have increased expression of extracellular matrix component or fibrosis genes with dysregulated fibroblast growth factor signaling. Hence, a severe sustained ( ~ 1/3 gestation) reduction in fetal left heart flow is sufficient to cause left heart hypoplasia. This is accompanied by changes in cellular composition and gene expression consistent with a pro-fibrotic environment and aberrant induction of mesenchymal programs.

## Abstract (web summary)

A fetal lamb model of mitral stenosis shows that sustained reduction in fetal left heart flow causes left heart hypoplasia and sequencing analysis identifies changes associated with pro-fibrotic environment and aberrant induction of mesenchymal programs.

## Introduction

Left ventricular (LV) hypoplasia is a component of many forms of congenital heart disease. In its most severe form, hypoplastic left heart syndrome (HLHS), all the left heart structures are diminutive and cannot support the systemic circulation. Despite reconstructive surgery, clinical outcomes for HLHS are not optimal: survival of 64% or 74% at 1 year^1^ and 59% or 64% at 6 years^2^, depending on the type of surgery. Only ~50% of patients with HLHS reach 18 years of age and by early adulthood face multiple challenges including heart failure^3^.

The etiology of LV hypoplasia remains uncertain and is likely to be heterogeneous. In some fetuses, LV hypoplasia may become apparent after the completion of cardiogenesis, marked by closure of the ventricular septum at 9.1 weeks of human gestation (Carnegie stage 22). In the specific context of HLHS, a post-cardiogenesis onset of LV growth failure is supported by the observation that an intact ventricular septum is found in the commonest anatomical pattern of HLHS (stenosis or atresia of the aortic and/or mitral valves)^4–6^, and that progression of severe fetal aortic stenosis to HLHS has repeatedly been documented in the second or third trimester of pregnancy^7^. The hemodynamic consequence of closure of the ventricular septum is that fetal LV filling becomes more precarious: it is entirely dependent on flow across the left atrioventricular valve and can no longer be compensated by flow across the ventricular septum.

Hemodynamic forces were hypothesized to play a role in the etiology of HLHS and animal models have demonstrated that blood flow affects the development and growth of cardiovascular structures. A 70% decrease in rabbit carotid artery flow over 2 weeks resulted in an endothelium-dependent decrease in diameter that was resistant to vasodilation^8^. In zebrafish embryos, ventricular inflow or outflow obstruction had profound effects on heart morphogenesis, including valve formation and chamber differentiation; a distal growth failure was attributed to lower shear forces on endocardial cells^9^. In early chick embryos, unilateral vitelline vein ligation resulted in structural malformations of the pharyngeal arch arteries and the heart^10–12^. The resultant cardiac defect, most commonly an abnormality of the ventricular septum or semilunar valves, depended on the extent of flow reduction^13^. In older chick embryos after cardiogenesis, inflow obstruction of the left heart resulted in diminished heart growth^14^.

In humans and other mammals, the basis for fetal cardiac growth is myocyte proliferation^15–18^ with a decline in mitotic index prior to birth^19^. The current understanding is that after birth, cardiomyocyte proliferation continues to slow and by adult life turnover is <1% per year of the total pool^20,21^. Hence, postnatal heart growth is mainly by an increase of the volume of individual cardiomyocytes (hypertrophy) and proliferation of interstitial tissue^20^. Quantifying the cellular composition of the heart from tissue sections requires stereology^15,17^ or dispersion of the heart followed by novel flow cytometry protocols^22^ (Table S1). Cardiomyocytes are particularly difficult to quantify due to their morphology, size, multinucleation, and lack of nuclear markers^23^. Single cell (or single nucleus) transcriptomics are powerful methods to study complex tissues at the single cell level and has enabled unprecedented insights into the cellular composition of developing hearts^24,25^. Single-cell RNA sequencing on induced pluripotent stem cells (iPSC) from patients with HLHS found abnormal endocardial signaling^26^ and intrinsic cardiomyocyte differentiation and maturation defects^27^.

A fetal mammalian model of HLHS using LV inflow or outflow obstruction was proposed in 1978^28^. LV inflow obstruction (LVIO) in fetal lambs led to early death within 2–7 days, with a 17% decrease in the ratio of left to right ventricular (RV) weight^28^. Supravalvar aortic banding was better tolerated than LVIO in these fetal lambs and autopsy measurements suggested LV hypoplasia^28^. In more recent studies, using echocardiography, fetal lamb supravalvar aortic banding did not consistently produce left heart hypoplasia but instead resulted in LV hypertrophy/dilation^29^. To date, attempts to create fetal lamb models of HLHS have been technically challenging.

To explore the pathogenesis of left heart growth failure in a large mammal, we decreased left heart flow by generating a fetal lamb model of mitral stenosis starting at 0.52 and ending at 0.84 gestation. From 0.51 to 0.97 gestation, the number of cardiomyocytes in the fetal lamb LV free wall increases approximately linearly from 0.6 to 2.4 × 10^9^ cells^30^. Between 0.75 and 0.84 gestation, 5–10% of cardiomyocytes are estimated to be active in the cell cycle, resulting in a 30% increase in cardiomyocyte numbers^31^. Hence, our fetal mitral stenosis model explores the effect of decreasing left heart flow during a period of cardiac growth by hyperplasia. Using single-nucleus RNA sequencing (snRNA-seq), we studied cell type composition, lineage inferences, and differential gene expression in hypoplastic LV myocardium.

## Results

### Preliminary experiments

**Fig. 1:**
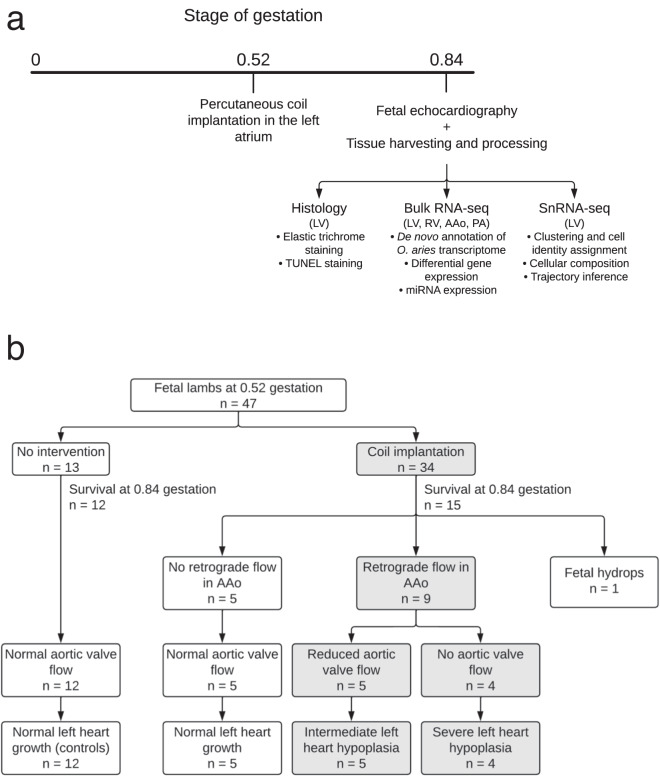
Fetal lamb model for hypoplastic left heart syndrome and experimental schema. **a** Outline of the study: coil implantation at ~76 days (0.52 gestation) and reassessed ~48 days later. Fetal echocardiography and tissue harvesting for histology, bulk, and snRNA-seq were performed at ~124 days (0.84 gestation). **b** Forty-seven fetal lambs were studied. Thirteen controls had no intervention (*n* = 12 survived). Thirty-four fetal lambs had coils implanted and 15 survived to 0.84 gestation: five had normal aortic valve flow and a normal left heart, nine fetuses had diminished aortic valve flow with compensatory retrograde flow in the ascending aorta and had developed left heart hypoplasia (non-apex forming left ventricles in four). Fetal hydrops in one fetus was secondary to coil-induced severe mitral regurgitation. The control fetus that died was the twin of a lamb that received coils. AAo ascending aorta, LV left ventricle, PA pulmonary artery, RV right ventricle.

Initial attempts to decrease left heart flow by filling the fetal LV with coils (*n* = 11) were unsuccessful as the lambs either died acutely or coil-induced ventricular tachycardia prevented implantation of a sufficient number of coils to decrease left heart flow. This was despite varying gestational age (65–121 days) at implantation, coil diameter, and the route of needle entry into the LV: directly through the LV wall or entry through the lumen of the mitral valve from the left atrium (LA). Since entry into the heart through the LA was better tolerated, we delivered a larger number of coils into the LA only and observed acute underfilling of the LV in a fetus at day 121 that died within one week. Having observed an acute effect, the approach of deploying coils wholly within the LA was repeated at a younger age (*n* = 2, 97 days): this was tolerated and at day 130, one fetus had retrograde flow in the AAo, a hypoplastic AAo as compared to the PA, and an underfilled but normal sized, apex-forming LV. This encouraged us to attempt coil implantation in the LA even earlier (~76 days, 0.52 gestation) and with the potential for a longer duration of low left heart flow (until ~124 days, 0.84 gestation; Fig. 1a). These fetal lambs are the subject of this report.

### Fetal lamb model of left heart hypoplasia

**Fig. 2:**
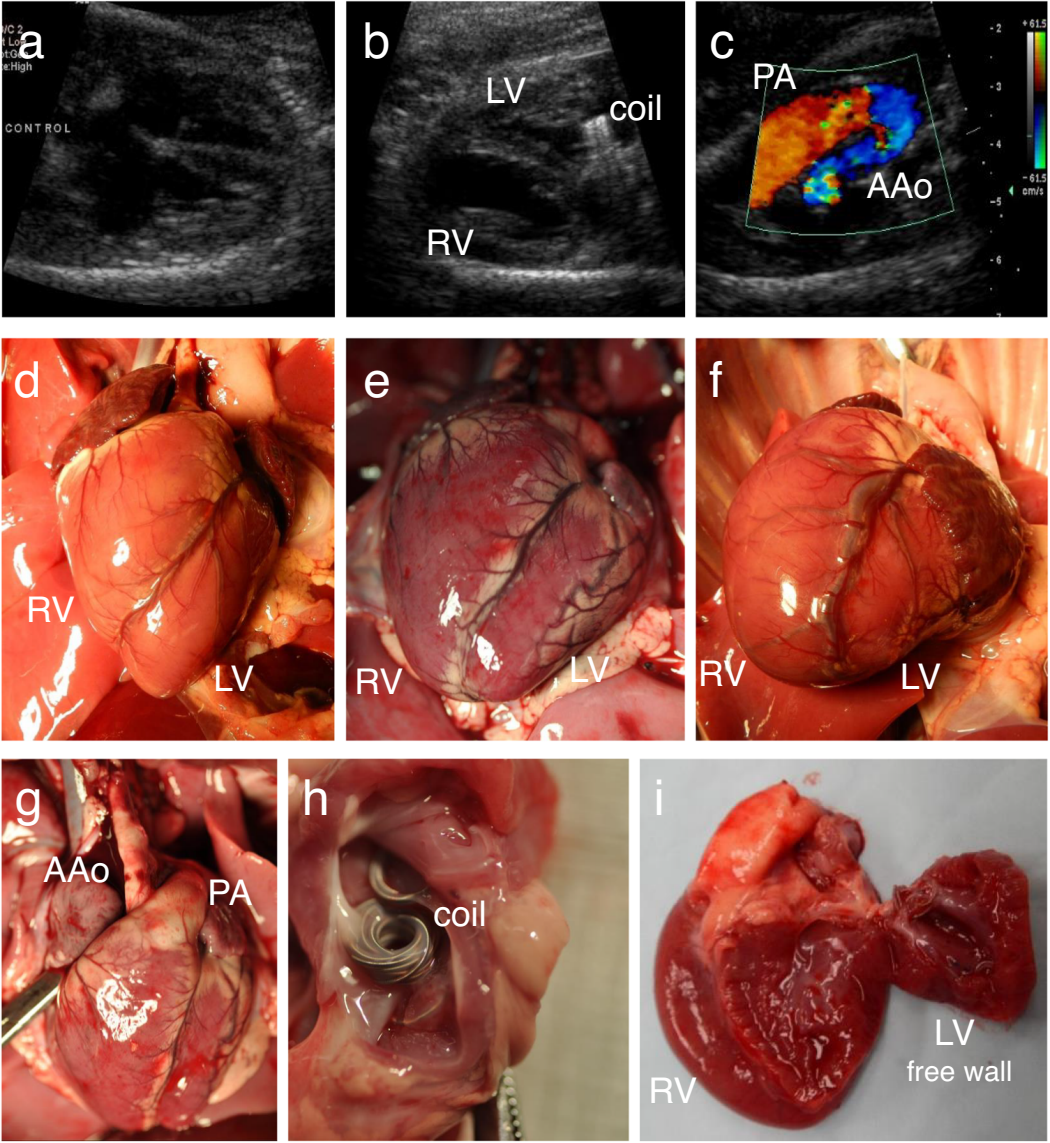
Left ventricular hypoplasia after atrioventricular flow obstruction in fetal lambs (0.84 gestation). Echocardiographic four chamber view of a control (**a**) and a hypoplastic left ventricle (**b**). **c** Retrograde flow in the ascending aorta. **d**–**g** Exposed hearts after a sternotomy. **d** Control with the left ventricle forming the apex; **e** Intermediate left heart hypoplasia with both ventricles forming the apex; **f** Severe left heart hypoplasia with the right ventricle forming the apex; **g** Intermediate left heart hypoplasia, with hypoplastic AAo. **h** Left atrium from above, with tissue growing into and covering the coils, creating left ventricular inflow obstruction in our model of mitral stenosis. **i** Severely hypoplastic left ventricle, left ventricular free wall opened. AAo ascending aorta, LV left ventricle, PA pulmonary artery, RV right ventricle.

Forty-seven lambs in 24 ewes were used (6 singleton, 13 twin, and 5 triplet pregnancies): 34 fetuses had left atrial coils implanted at 76 (IQR: 75, 80) days or 0.52 (IQR: 0.51, 0.54) gestation, and 13 were non-instrumented controls (Fig. 1b and Table S2). Nineteen out of 34 (56%) coiled fetuses died secondary to instrumentation and acute circulatory change. Twelve (92%) controls and fifteen (44%) coiled fetuses survived the 46 (IQR: 45, 48) days or 0.31 (IQR: 0.31,0.33) gestation to echocardiographic assessment at 124 (IQR: 123, 125) days or 0.84 (IQR: 0.84, 0.85) gestation (Figs. 2a, b and S1). One of the 15 surviving coiled fetuses was severely hydropic (fetal weight of 10 kg), secondary to coil-induced severe mitral regurgitation and was not included in further analyses.

In the remaining 14 coiled fetuses, the severity of LVIO was assessed by the presence of retrograde flow across the ascending aorta^32^ compensating for deficient antegrade flow across the aortic valve (Figs. 2c and S1). Fetuses with (i) no/minimal LVIO had normal antegrade flow across the aortic valve and no retrograde flow in the AAo (*n* = 5), while those with (ii) LVIO had retrograde flow in the AAo (*n* = 9; Fig. 2c). Five of the fetuses with retrograde flow in the AAo had intermediate LVIO with some remaining aortic valve flow, and four had severe LVIO, with no antegrade aortic valve flow, and only retrograde perfusion of the brain and coronary arteries from the arterial duct.

Echocardiography and autopsy confirmed that the five coiled hearts with normal antegrade flow across the aortic valve either had very few or no coils within the left atrium (likely due to early coil embolization out of the heart), or the coils lay far from the mitral valve in the posterior left atrium/left atrial appendage and produced no LVIO. All nine fetuses with LVIO displayed LV growth failure. Hearts with the most severe LVIO (*n* = 4) had left heart hypoplasia including non-apex forming LVs (Fig. 2f). Those with intermediate degrees of LVIO (*n* = 5) had small left hearts, but with less severe growth failure e.g., the LV still sharing the apex with the RV (Fig. 2e).

Coiled and control fetuses were from singleton to triplet pregnancies, resulting in considerable variation in size. Their weight ranged from 1.74 to 5.12 kg (controls: 3.0 ± 0.78 kg, coiled with retrograde AAo flow: 3.28 ± 0.68 kg; *p* = 0.39; Table S2), excluding the hydropic fetus. Due to the large fetal size range, left heart measurements were normalized to the corresponding right heart structures or the total heart weight. LV free wall weight (−39% mean), LV end-diastolic dimensions (diameter: −52% mean; length: −29% mean), and diameters of the aortic valve (−38% mean) and AAo (−30% mean) were significantly reduced in the low-flow LVs as compared to controls (Table 1). Our model demonstrated a flow-dependent growth failure of the fetal left heart, which recapitulated hallmarks of human HLHS, including retrograde perfusion of the brain and coronary arteries from the arterial duct.

**Table 1:** Cardiovascular measurements (mean ± standard deviation) in fetal lambs, at 0.84 gestation.

	Controls (*n* = 12)	Retrograde AAo flow (*n* = 9)	*p* value
Aortic / pulmonary valve diameter^a^	0.87 ± 0.12	0.54 ± 0.11	0.00015
AAo / PA diameter^a^	0.83 ± 0.14	0.58 ± 0.07	0.00036
LV / RV end-diastolic diameter^a^	1.07 ± 0.09	0.49 ± 0.16	0.00002
LV / RV end-diastolic length^a^	1.16 ± 0.09	0.82 ± 0.21	0.00041
LV free wall / total heart weight^b^	0.23 ± 0.04	0.14 ± 0.01	0.008

Due to fetal size variations, left heart echocardiographic measurements were normalized to the corresponding right heart structures. The weight of the LV free wall was normalized to the total heart weight. Mitral valve diameter could not be measured due to ultrasound scatter from the coils. Sample size: *n* = 12 biologically independent controls, *n* = 9 biologically independent coiled lambs.

*AAo* ascending aorta, *LV* left ventricle, *PA* pulmonary artery, *RV* right ventricle.

^a^Echocardiographic measurements.

^b^Autopsy measurements.

**Fig. 3:**
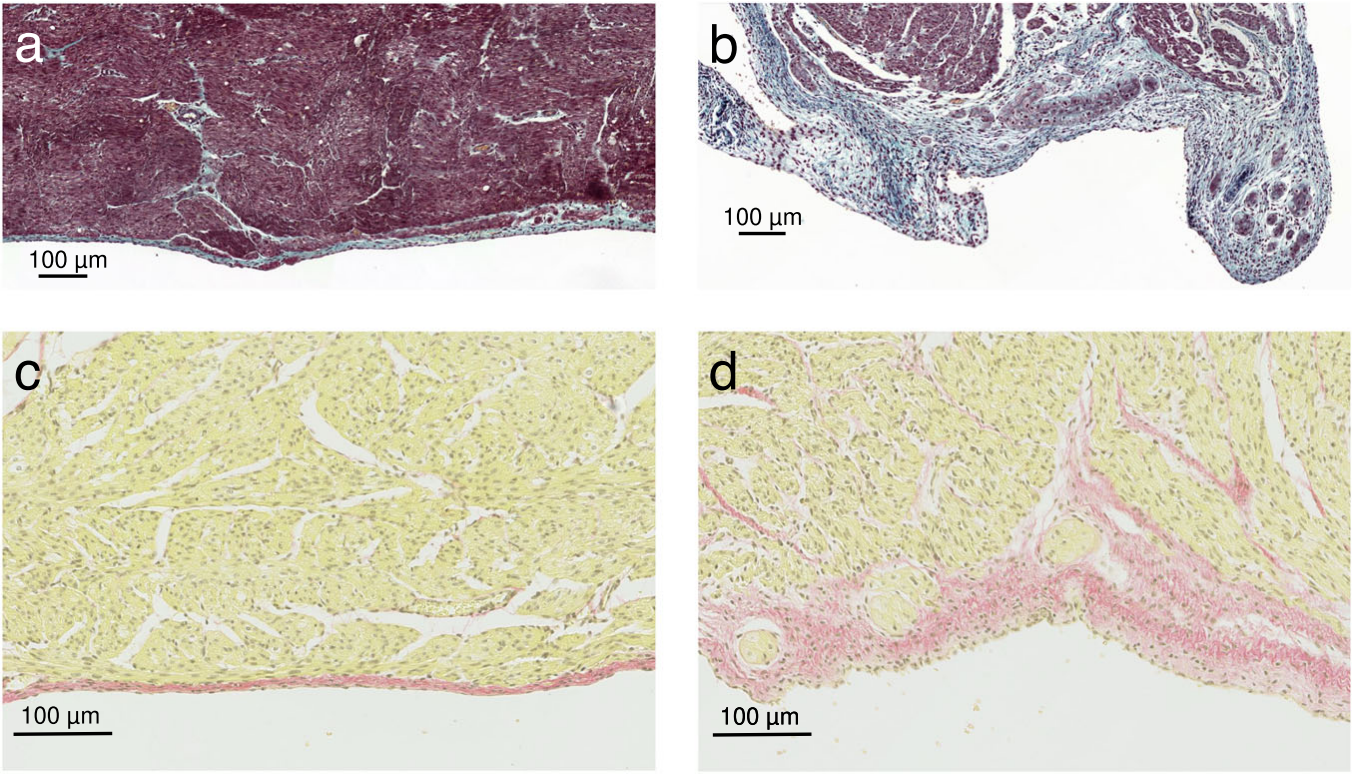
Histology of the endocardial layer of ovine fetal left ventricular free-wall tissue (0.84 gestation). The thicker endocardium of hypoplastic left ventricles has increased collagen staining (**a**, **b**, blue; **c**, **d**, red). Elastic trichrome staining: **a** Control; **b** severe left heart hypoplasia. Picro-Sirius Red staining: **c** control; **d** severe left heart hypoplasia.

In the hypoplastic LVs, as compared to controls, the endo/subendocardial layer varied in thickness but was thicker throughout (*p* = 0.015) and had prominent Purkinje fibers in elastic-trichrome stained sections (Figs. 3, S2 and S3). The subendocardial layer of the myocardium from hypoplastic LVs was less densely packed and edema may have contributed to the thickening. There was no evidence of endocardial elastosis, inflammation, or increased fibrosis in the myocardium from the hypoplastic LVs, when examined by a clinical pathologist and confirmed by Picro-Sirius red staining, however there were various regions with increased subendocardial connective tissue staining in coiled tissues (Figs. 3 and S4). Apoptosis as assessed by TUNEL assays was not found in either hypoplastic or control ventricles (Fig. S5).

### Bulk RNA sequencing of hypoplastic left ventricles and aortae

We performed bulk RNA sequencing primarily to enable a de novo annotation of the *O. aries* transcriptome to improve mapping and annotation of the snRNA-seq data. We also assessed differential mRNA and miRNA expression in AAo (*n* = 4 hypoplastic, *n* = 2 controls) and LV (*n* = 4 hypoplastic, *n* = 4 controls). We observed high correlation between biological replicates (samples from the same tissue and condition; >0.88 for AAo and >0.91 for LV). We also noticed between-sample variation, which may outweigh effects from the coiling for some samples (Fig. S6) and may be related to some samples having low RNA integrity numbers (RIN). After removing outliers, we identified 64 significantly upregulated, and 85 downregulated genes in AAo (Figure S7a; upregulation refers to higher expression in coiled fetuses). Pathway analyses of significantly differentially expressed genes revealed enrichment for cellular components of the cell periphery (*p* = 0.022) and plasma membrane (*p* = 0.029; Supplementary Data 1). Additionally, we identified 4 upregulated miRNAs in AAo (Fig. S8a). For three of those miRNAs, we found significantly downregulated target genes (Fig. S8b), including genes involved in cell growth and proliferation (*DDIT4*, *HS6ST2*) and cell adhesion (*NRXN3*, *IGFS3*, *HS6ST2*). For the LV, we discovered 4 significantly upregulated, and 13 downregulated genes (Fig. S7b), enriched for brown fat cell differentiation processes. We also identified 4 upregulated and 7 downregulated miRNAs (Fig. S9a). Among the top upregulated miRNAs was *miR-15a-5p*. In mice, overexpression of miR‐15 family members was associated with cardiomyocyte cell cycle withdrawal and smaller heart size^33^. Two upregulated miRNAs had significantly downregulated target genes, and three downregulated miRNAs had significantly upregulated target genes (Fig. S9b). Additional “novel” miRNAs in AAo and LV could not be matched with their respective orthologues and were therefore not evaluated. While the low RIN values necessitate cautious interpretations, we found differentially expressed miRNAs and target genes associated with cell growth, proliferation and adhesion. However, we could not attribute changes to specific cell types or biological pathways.

### SnRNA-seq of hypoplastic left ventricles

**Fig. 4:**
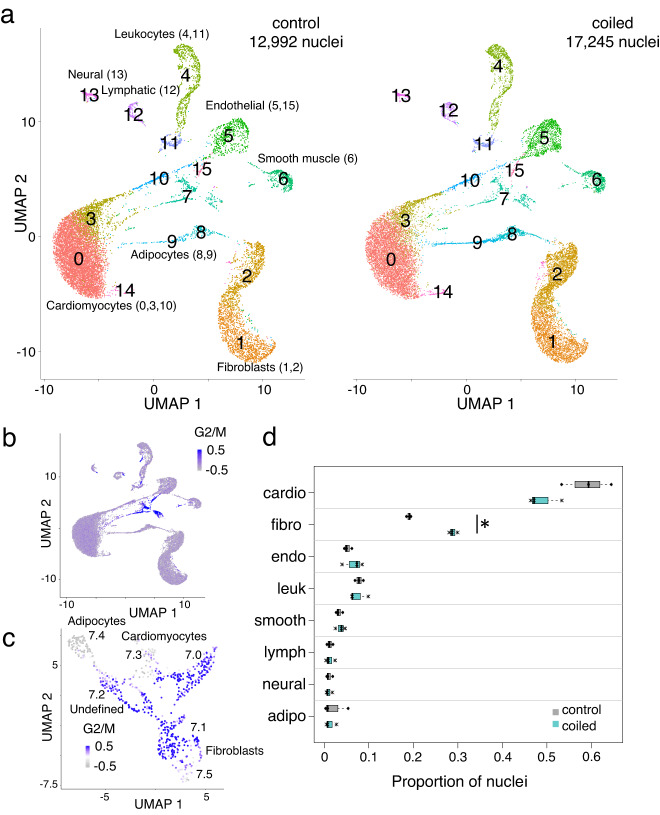
Single nucleus RNA sequencing revealed clusters of all major cardiac cell types, with subsets of nuclei expressing cell cycle related genes. **a** UMAP plots of nuclei from control (left) or coiled (hypoplastic) left ventricles (right). Clusters were assigned to the major cardiac cell types: adipocytes (8, 9), cardiomyocytes (0, 3, 10), endothelial cells (5, 15), fibroblasts (1, 2), leukocytes (4, 11), lymphatic endothelial cells (12), neural cells (13), and smooth muscle cells (6). **b** Cluster 7 expressed cell cycle related genes (G2 phase, mitosis). **c** Cluster 7 nuclei were re-clustered and displayed with new coordinates: adipocytes (7.4), cardiomyocytes (7.0, 7.3), fibroblasts (7.1, 7.5), undefined (7.2). **d** Boxplots (proportions of nuclei: median and interquartile ranges) in coiled (hypoplastic) and control left ventricles, showing a significant increase in the proportion of fibroblasts (*) and a non-significant decrease in cardiomyocyte nuclei. Numerical data in Table S5. Sample size: *n* = 3 biologically independent controls, *n* = 3 biologically independent coiled lambs.

To further evaluate changes in the myocardial cellular composition and cell type specific differential gene expression, we performed snRNA-seq on LV free wall samples. We captured 17,245 nuclei from coiled, severely hypoplastic left hearts (*n* = 4 fetuses with no or minimal flow across the aortic valve and no mitral regurgitation) and 12,992 nuclei from controls (*n* = 3 non-instrumented fetuses; Fig. S10). We identified three clusters of cardiomyocytes (0, 3, 10), two clusters of fibroblasts (1, 2), one cluster of smooth muscle cells (6), two clusters of endothelial cells (5, 15), one cluster of lymphatic endothelial cells (12), two clusters of adipocytes (8, 9), two clusters of leukocytes (4, 11), and one cluster of neural cells (13) (Figs. 4a and S11). No cell types were unique to either hypoplastic or control myocardia (Table S3). Within cell types, there was marked heterogeneity in gene expression. Compared to the other cardiomyocyte clusters, cluster 0 enriched for actin binding structures (actinin, z-discs, cell-cell adherens junctions) indicating a role in mechanical stability, while cardiomyocyte clusters 3 and 10 enriched for genes involved protein biosynthesis. These cardiomyocyte clusters may represent different stages of cardiomyocyte maturation^34^. Within fibroblasts, cluster 1 enriched for genes involved in protein biosynthesis including traditional fibroblast markers, such as extracellular matrix components. Cluster 2 enriched for ion channels, and might represent a subtype of fibroblasts predominantly involved in cellular communication^35^.

Clusters 7 and 15 were enriched for G2 phase/mitosis-related genes (Fig. 4b) suggestive of high cell cycle activity. They constituted only a small percentage of the total number of nuclei, with no obvious difference between experimental conditions (cluster 7: control 2.8%, coiled 2.3%; cluster 15: control 0.3%, coiled 0.3%. Wilcoxon’s test between the ratio of cell types did not identify significant differences). Marker gene expression analyses identified cluster 15 as endothelial cells. As we could not assign the entire cluster 7 to one of the major cardiac cell types, we performed sub-clustering (Fig. 4c) and identified cycling cardiomyocytes (clusters 7.0, 7.3), cycling fibroblasts (clusters 7.1, 7.5), and cycling adipocytes (cluster 7.4; Fig. S12 and Table S4). Notably, the number of cycling cardiomyocytes was consistent with Ki67 staining in the literature^16^.

Next, we assessed whether the myocardium from hypoplastic LVs only differed in quantity, or whether the composition had changed. Large mammals have prominent focal collections of epicardial adipocytes that can render myocardial samples unrepresentative; one coiled LV sample was excluded from compositional analysis as it had a high proportion of adipocytes (Table S5 and Fig. S13). We analyzed the cellular composition by the proportion of each cell type in control and hypoplastic LV samples by two methods: a Fisher’s exact test (Fig. S14 and Table S6) and Dirichlet-multinomial logistic regression (Bayesian *scCODA*^36^ and frequentist *Dirichlet Reg*^37,38^ (Table S7). All analyses found a significant increase in fibroblasts. Both *scCODA* and *DirichletReg* found a ~ 1.4 -fold increase in fibroblasts, corresponding to 20.1% of nuclei in controls vs. 28.7% in hypoplastic myocardia (Fig. 4d and Table S7). In contrast, the fraction of cardiomyocyte nuclei was decreased in the hypoplastic myocardia (59.1% vs. 52.7%; Fig. 4d). This decrease was significant by the FET, but not by *scCODA* or *DirichletReg*, likely due to the greater sample variability in cardiomyocyte counts (Table S3).

**Fig. 5:**
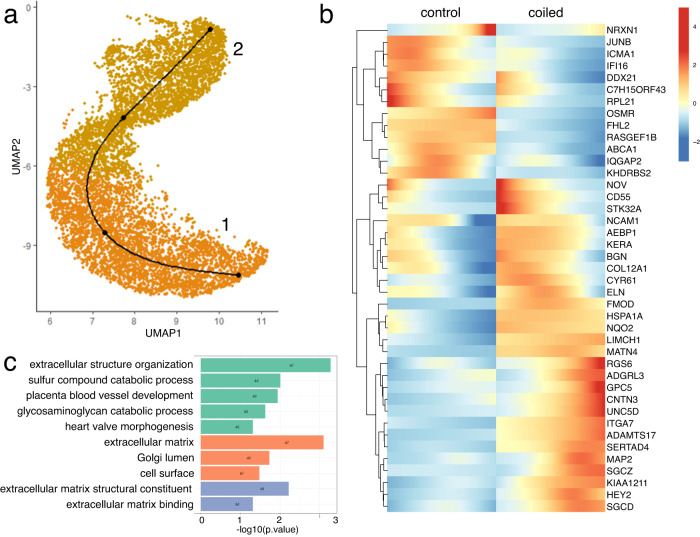
Fibroblast lineage inference and differential expression analysis. **a** Fibroblast manifold (controls and coiled samples pooled). **b** Heatmap representing differentially expressed genes over pseudotime, controls (left) and coiled (hypoplastic) left ventricles (right). **c** Gene ontology (GO) enrichment analysis of differentially expressed genes. Sample size: *n* = 3 biologically independent controls, *n* = 4 biologically independent coiled lambs.

**Fig. 6:**
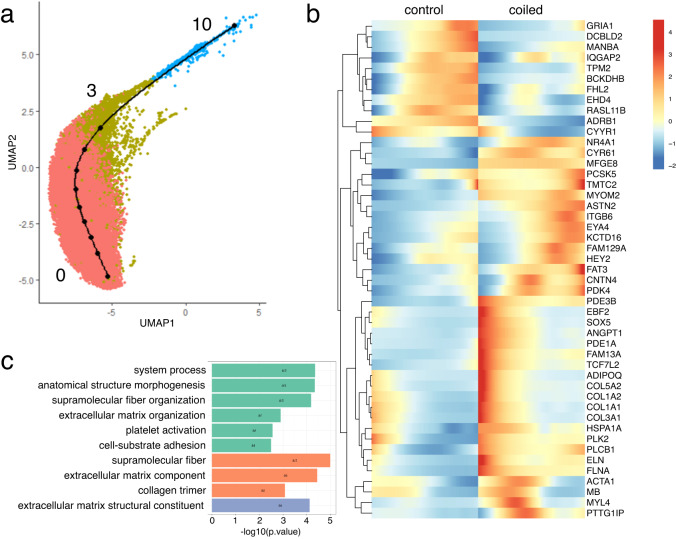
Cardiomyocyte lineage inference and differential expression analysis. **a** Cardiomyocyte manifold (controls and coiled samples pooled). **b** Heatmap representing differentially expressed genes over pseudotime, controls (left) and coiled (hypoplastic) left ventricles (right). **c** Gene ontology (GO) enrichment analysis of differentially expressed genes. Green: biological process; Orange: cellular component; Blue: molecular function. Sample size: *n* = 3 biologically independent controls, *n* = 4 biologically independent coiled lambs.

Although we studied a single time-point in development (0.84 gestation), each “cell type” is composed of a spectrum of cellular states some of which are transient. We studied the dynamics of gene expression by lineage inference for the most abundant cell types: fibroblasts, cardiomyocytes and endothelial cells (Figs. 5, 6, S15, S16, and S17). Cell cycle active fibroblast and cardiomyocyte subclusters were excluded from our pseudotime analysis because cell-cycle regression analysis suggested that these were cycling mature cells, rather than a manifold differentiating into mature cell types. Pseudo-timepoints along the fibroblast manifold were associated with response to TGF-beta signaling, external stimuli, and chemicals (Supplementary Data 2 and Fig. S15). Comparing fibroblasts from hypoplastic versus control myocardia, we identified differentially expressed genes along the pseudotime axis. Genes upregulated in hypoplastic left hearts were involved in the organization and composition of the extracellular matrix (*ELN*, *COL12A1*, *FMOD*, *MATN4*, *ITGA7*, *BGN*, *AEBP1*, *KERA*, *GPC5*), and in heart valve morphogenesis (*ELN*, *HEY2*, *CYR61*). Downregulated genes were associated to the cell surface (*IQGAP2*, *ABCA1*; Fig. 5 and Supplementary Data 2). Similar pathways were identified when differential gene expression was analyzed by cluster (Supplementary Data 3). The cardiomyocyte manifold was associated with cell proliferation and growth regulation, electrical coupling, and actin-mediated cellular contraction (Supplementary Data 2 and Fig. S16). Cardiomyocytes from hypoplastic left hearts expressed higher levels of extracellular matrix components (*ELN*, *COL1A1*, *COL1A2*, *COL3A1*, *COL5A2*, *ITGB6*, *MFGE8*) and genes involved in morphogenesis (*MYOM2*, *ELN*, *COL1A1*, *EYA4*, *HEY2*, *MFGE8*, *CYR61*, *CNTN4*, *ASTN2*, *ANGPT1*, *COL1A2*, *COL3A1*, *ADIPOQ*, *FLNA*). Upregulated genes were also involved in transforming growth factor (TGF)-beta signaling (*COL1A1*, *COL1A2*, *COL3A1*, *SOX5*) and integrin signaling (*ITGB6*, *MFGE8*, *CYR61*, *COL3A1*; Fig. 6 and Supplementary Data 2). We also found dysregulation of genes related to cardiac contraction, such as *TPM2* downregulation and *MYL4* upregulation. *MYL4* expression (encoding a fetal myosin light chain) can improve myocardial contractility and was found in a variety of congenital heart diseases and cardiomyopathies^39,40^. Inference of the endothelial lineage revealed association of genes related to signal transduction, cell migration, growth, and extracellular matrix binding. Differential gene expression suggested upregulation of extracellular matrix constituents (*VCAN*, *ELN*, *FN1*, *TNXB*) and genes associated with glycosaminoglycan binding (*VCAN*, *FN1*, *SLIT2*, *TNXB*; Supplementary Data 2 and Fig. S17). Hence, extracellular matrix components were upregulated in all three cell types, suggesting an induction of mesenchymal programs (Fig. S18).

**Fig. 7:**
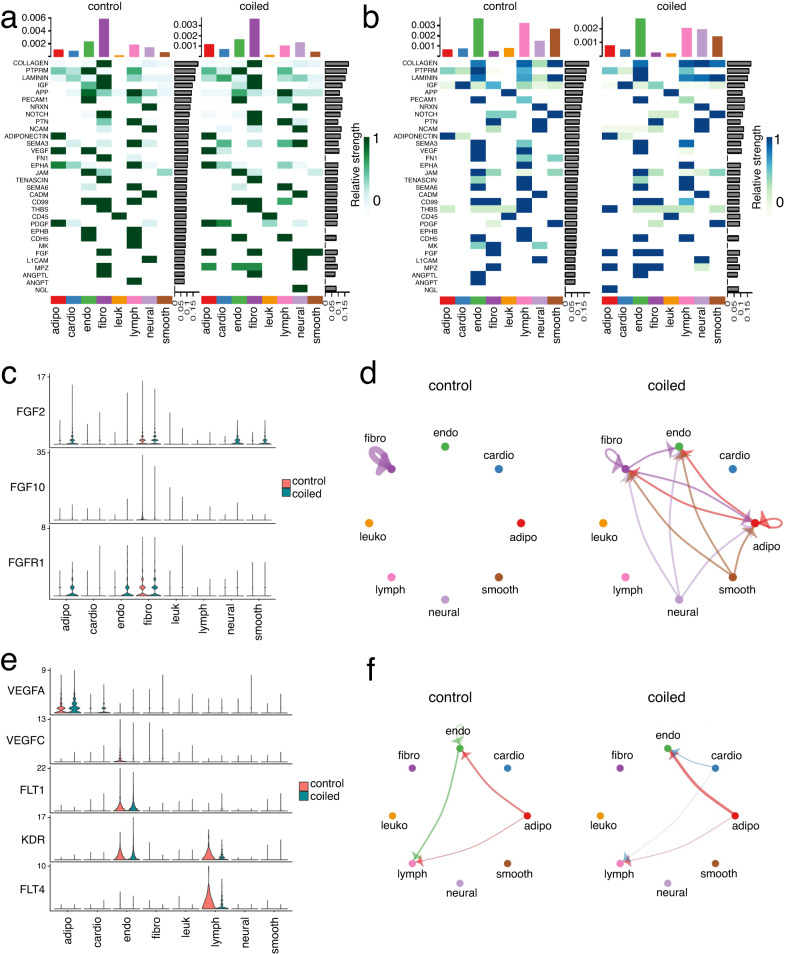
Cellular communication networks from single nucleus RNA sequencing data. Cellular communication was compared between “control” and “coiled” LV samples using CellChat’s four network centrality measures. Heatmaps of communication intensity by signaling molecule (rows) and cell-type (columns) showing outgoing (**a**) and incoming (**b**) patterns. Marginal barplots represent total signaling strength for each cell-type (column-associated) or signaling pathway (row-associated). Violin plots of normalized gene expression within the fibroblast growth factor (FGF) pathway (**c**), and the VEGF pathway (**e**), biologically relevant coiled-upregulated signaling pathways that also contains coiled-specific differentially regulated ligand-receptor pairs. Circle plots modeling communication within the FGF pathway (**d**), and the VEGF pathway (**f**), across different cell types. Sample size: *n* = 3 biologically independent controls, *n* = 4 biologically independent coiled lambs.

CellChat is an exploratory analysis, identifying differentially regulated ligand-receptor pairs by cell type. Using CellChat, we predicted differences in cellular communication between control and coiled. We detected 32 enriched signaling pathways among the 8 cell groups, including Collagen, IGF, FGF, NOTCH, and VEGF signaling (Fig. 7a, b). While the communication network patterns were similar in controls and hypoplastic samples, output signals were overall stronger in control fibroblasts (Fig. 7a). Differential communication of ligand-receptor interactions suggested alterations in fibroblast growth factor (FGF) and vascular endothelial growth factor (VEGF) signaling. FGF receptor *FGFR1* had higher expression in endothelial cells from hypoplastic LV, while *FGF2* was found upregulated in adipocytes and endothelial cells (Fig. 7c, d). VEGF receptor expression was dysregulated in lymphatic (*FLT4;* VEGFR3) and endothelial cells (*KDR*; VEGFR2), while its ligand VEGFA was upregulated in cardiomyocytes from hypoplastic myocardium (Fig. 7e, f). Further evidence was provided by differential analysis using MAST (Supplementary Data 3).

Overall, our lamb model of LVIO suggests that mid-gestation growth of left heart structures is flow-dependent. The snRNA-seq data indicates an increased fraction of fibroblasts, and an upregulation of genes related to extracellular matrix and tissue remodeling in various cell types. Cellular communication modeling suggested alterations in FGF and VEGF signaling. These findings were associated with a thicker subendocardial layer of connective tissue but no global fibrosis, which was appropriate for this stage of prenatal development.

## Discussion

We created an experimental model of HLHS by implanting coils in the left atrium of fetal lambs to gradually reduce blood flow into the LV at mid-gestation, long after cardiogenesis was complete. The model recapitulated important clinical features of fetal HLHS, including decreased antegrade aortic valve flow, retrograde perfusion of the brain, severe left heart hypoplasia, and a non-apex forming LV. The myocardium of hypoplastic LVs had no overt fibrosis but a thickened subendocardial layer of connective tissue, and transcriptomic analyses revealed quantitative and qualitative changes in cellular composition, gene expression, and intercellular signaling networks.

The interval spanned by our fetal lamb experiments (0.52–0.84 gestation) corresponds to 20–34 weeks of human gestation. Human fetal aortic stenosis has been observed to progress to HLHS during this period^7^, which is predicted by the presence of retrograde flow in the transverse aortic arch^32^. The severity of flow disturbance in our model can be estimated for the AAo: normal antegrade flow through the AAo and coronary arteries are 35–40% and ~4% of the combined ventricular output, respectively. In fetuses with no antegrade AAo flow, the AAo is only a conduit for the coronary arteries and hence has only one tenth of the normal flow volume in the opposite direction^41^. Such a severe and sustained decrease in LV inflow was sufficient to cause reduced growth of downstream structures, resulting in non-apex forming LVs, with small aortic valves and AAo (Fig. 2 and Table 1).

The cellular and molecular basis for cardiovascular growth is an active area of research. From bulk RNA sequencing, some of the differentially expressed miRNAs were associated with cell proliferation and cell cycle withdrawal, e.g., *miR-15a-5p*^33^. However, from the pooled gene expression of thousands of cells of differing types and states, we were unable to infer a clear global myocardial tissue response to low left heart flow. We therefore used snRNA-seq to investigate the response of distinct myocardial cell types in terms of changes in their population and their transcriptomes^36^. The major change in cellular composition was a ~1.4-fold increase in the proportion of fibroblasts from 20.1% (controls) to 28.7% (coiled) of nuclei, which was consistent amongst all samples. Since proportions sum to unity, if one cell type increases in proportion there is an obligate decrease in others. In our hypoplastic LV myocardium, this was restricted to a decrease in cardiomyocyte nuclei (Fig. 4d) from 59.1% (controls) to 52.7% (coiled). Thus, LV growth failure in our LVIO model was associated with a change in cell type fractions, implying the mechanical signals of blood flow and diastolic preload had a differential effect on the proliferation and/or differentiation of fibroblasts and cardiomyocytes. The transcriptomes of hypoplastic ventricles at 0.84 gestation (75 days after the onset of LVIO) reflect tissue remodeling processes rather than early-response signaling pathways. Multiple cell types, including fibroblasts, cardiomyocytes, and endothelial cells were found to have increased expression of extracellular matrix components (Fig. S18). Inference of known ligand-receptor groups and modeling of cellular communication networks predicted altered signaling within FGF and VEGF pathways (Fig. 7)^42–45^. An upregulation of collagens or other extracellular matrix proteins in endothelial cells is one of the hallmarks of endothelial to mesenchymal transition (EndMT), and both FGF2 signaling through FGFR1, and VEGF-A signaling through VEGFR2 have been found protective or inhibitory against EndMT^46–49^. Hence, hypoplastic LVs presented with features that were suggestive of an aberrant and ectopic induction of mesenchymal and pro-fibrotic signaling, including upregulated expression of extracellular matrix components, an increased abundance of fibroblasts, and dysregulations of FGF and VEGF signaling as potential anti-EndMT responses (Supplementary discussion).

Consistent with those cellular and molecular changes, hypoplastic myocardia had a demonstratively thicker layer of subendocardial connective tissue. In children with HLHS, a thick endocardial layer of cellular fibroelastic tissue, termed endocardial fibroelastosis (EFE), was associated with pathological EndMT and was considered predictive of clinical outcomes, for example after fetal interventions^50,51^. While in this fetal lamb model of HLHS there was no severe fibrosis in histology, most of the cardiac collagen matrix develops postnatally^52,53^ . A fibrotic response may therefore become more apparent after birth or if pressure-overload is superimposed in fetal life, e.g., by aortic stenosis. Notably, subendocardial thickening and fibrosis with collagen deposition was observed in the chick embryo left atrial ligation model of HLHS^54^, and intrinsic endocardial defects and abnormal EndMT were found in human HLHS iPSC^26^. Even subtle enhancements of the collagen mesh between and parallel to myocytes^52,53^ could increase myocardial stiffness and limit growth by tempering the transduction of mechanical signals and acting as a physical constraint on cardiomyocyte lengthening and growth.

The demonstration of left heart hypoplasia secondary to low left heart flow does not undermine the importance of a genetic predispositions to HLHS and structural left heart disease^55^. Rather, it demonstrates the impact of hemodynamic forces in the translation of genotypes to phenotypes, resulting in some of the most clinically devastating morphological features of HLHS. The complexity of the cascades that translate genetic variation to a clinical disease are reflected in the heterogeneous genetic architecture of left heart malformations. A wide range of de novo and rare inherited variants contribute to the clinical spectrum, including various genes associated with EndMT (*FGFR2*, *FOXP1*, *GATA4*, *GJA1*, *NKX2-5*, *NOTCH1*, *NR2F2*, *SMAD3*)^27,56^. Other genetic predispositions may result in structural or functional cardiac abnormalities that will inevitably change left heart flow, which in turn results in morphological changes to the growing heart, representing a form of interaction between genes and the internal environment. Transduction of mechanical signals, flow and diastolic preload, may also be vulnerable to allelic variation. A complex etiology is exemplified in the digenic inheritance (*SAP130*, *PCDHA9*) of left heart hypoplasia with a ventricular septal defect in the Ohia mouse^57^ and reinforced by the occurrence of right heart rather than left heart disease (tricuspid valve dysplasia/atresia) and extra-cardiac abnormalities in pigs edited to be *SAP130* deficient^58^.

Sheep are the preferred species for studies of fetal mammalian physiology, especially if instrumentation is required. However, they impose significant constraints due to their reproductive seasonality, expense, limited molecular toolkit and range of reagents e.g., antibodies or annotated genomic assemblies^16,30^. The initial findings from this model immediately raise questions which we will address in future studies. For example, is there a developmental window during which flow perturbations have an effect and does this vary between left heart structures? We believe flow effects will have their largest impact whilst there is still rapid cardiomyocyte proliferation until ~0.75–0.9 gestation^16,30^. Once the perinatal slowing of cardiomyocyte proliferation occurs, there is little potential for catch-up growth by cardiomyocyte hyperplasia, and growth by hypertrophy may be limited by fibrosis. The development of novel agents and interventions for HLHS requires a mechanistic and molecular understanding of left heart hypoplasia. Large mammal models of left heart hypoplasia will be essential for preclinical evaluation of treatments, whether they be surgical, interventional catheterization or drug therapies. This includes the exploration of agents to promote cardiomyocyte proliferation, and to overcome the reduced physiological flow stimulus and fibrotic constraints. Our model recapitulates the flow patterns of human fetal HLHS with retrograde perfusion of the brain and coronary arteries from the arterial duct, and it will also allow an evaluation of the impact of this physiology on the development of other organs including the brain.

The gradual onset of severe left ventricular inflow obstruction at mid-gestation resulted in a severe sustained decrease in left heart flow, which was *sufficient* to result in unequivocal left heart hypoplasia. This implies that physiological fetal mammalian left heart growth after cardiogenesis is flow-dependent. Myocardium exposed to low blood flow had an increased fraction of fibroblasts and an upregulation of extracellular matrix and tissue remodeling genes in multiple cell types, suggestive of ectopic mesenchymal-like signaling. A susceptibility to pro-fibrotic prenatal tissue remodeling could further affect cardiac growth and function, including the outcome of fetal and early postnatal interventions.

## Methods

### Coil implantation in fetal lambs

We have complied with all relevant ethical regulations for animal testing. All procedures followed the Canadian Council on Animal Care guidelines and were approved by the University of Western Ontario Council on Animal Care (protocol 2010-257). Time-dated pregnant Dorset × Rideau Arcott ewes (gestational age 76 days, term 147 days) were fasted (16 h solids, 6 h water) and premedicated with ketoprofen (3 mg/kg; Anafen, Boehringer Ingelheim). Anesthesia was induced with 7–20 mg/kg intravenous (IV) sodium pentothal (typically ~ 1 g into an internal jugular vein; Abbott Laboratories Ltd, Montreal, Canada) and maintained with 1–3% isoflurane and 5–6 L/min O_2_. Ewes were monitored by heart rate, blood pressure, pulse oximetry and end-tidal capnography. Ultrasound was then used to determine litter size, and if multiples were detected, to decide which fetus had the best lie for coil implantation.

Coils were implanted in the left heart under continuous ultrasound guidance (Philips HDI 5000), using a completely percutaneous approach under strict aseptic conditions. This technique was adapted from that used in our clinical practice for human fetal cardiac interventions^59^. A 20 G needle (6” Quincke; Beckton-Dickinson) was used to deliver platinum coils (0.018” IDC, Boston Scientific) above the mitral valve until the LV appeared underfilled and the ascending aortic flow decreased or became retrograde. Antibiotic prophylaxis was given for 3 days after the procedure (trimethoprim-sulfadoxine; 16 mg/kg i.m. Trivetrin, Schering-Plough). Further information on sex and age of the fetuses in Table S2.

### Echocardiography

Forty-five to fifty days after coil implantation (0.84 gestation) ewes were anesthetized and fetal echocardiograms were performed to measure LV and RV end-diastolic and end-systolic dimensions, diameters of the aortic valve, pulmonary valve, ascending aorta (AAo) and main pulmonary artery (PA; Table S2). Color-flow mapping was used to evaluate mitral valvar, aortic, ductal, and atrial septal flow characteristics.

### Fetal lamb tissue handling

Under maternal general anesthesia and after fetal echocardiography, fetal lambs were delivered by hysterotomy. The umbilical cord was clamped and the lamb was euthanized with IV sodium pentobarbital (BimedaMTC, Cambridge, Canada). A median sternotomy was used to harvest the heart, then samples were taken from the fetal left ventricles (LV), right ventricles (RV), AAo and main PA. Tissue was either snap frozen with liquid N_2_ and stored at −80 °C (transcriptome analyses) or fixed in 4% formalin (histology). The ewe was euthanized with pentobarbital once all fetuses had been removed.

### Histology

Left ventricular myocardial samples were obtained from fetal lambs (*n* = 4 severely hypoplastic left hearts, *n* = 7 controls). After fixation in 4% formalin for 24 h, tissue was transferred to fresh phosphate-buffered saline (PBS) for 3 days, 70% ethanol for 7 days, then blocked, and embedded into paraffin. Stainings were performed at the STTARR facility (Innovation Centre for Advanced Preclinical Imaging and Radiation Research), Toronto, Canada (Elastic trichrome and TUNEL) or at The Centre for Phenogenomics, Toronto, Canada (Picro-Sirius Red). Elastic trichrome staining was used to assess histology and endo-/subendocardial thickness. Apoptosis was evaluated using the TUNEL assay. Connective tissue and collagen deposition was assessed by Picro-Sirius Red staining.

### Histology stainings

#### Elastic trichrome

Elastic trichrome staining is a combination of Verhoeff elastic stain and Masson trichrome, used to differentiate collagen from smooth muscle and demonstrate elastic fibers. Serial sections of 4–5 μm thickness were cut in transverse plane, deparaffinized and rehydrated in xylene (3 × 2 min), 100% and 95% ethanol, and water. Sections were incubated in mordant Bouin solution (Polysciences Inc) for 1 h at 56° Celsius, washed in water for 10 min, then stained in Verhoeff elastic stain (see below) for 1 h, and washed in distilled water. Sections were differentiated microscopically in 2% ferric chloride until elastic fibers were distinct and the background colorless to light gray. Sections were stained in Biebrich scarlet and acid fuchsin solution for 2 min, rinsed in distilled water, placed in 5% phosphotungstic acid solution for 15 min, stained with 2% light green solution for 10 min, placed in 1% acetic acid solution for 30 s, dehydrated with 95% and 100% ethanol + xylene, and then mounted with synthetic mounting media. Verhoeff elastic stain: Alcoholic hematoxylin, 5% (30 ml), Ferric chloride, 10% solution (12 ml), Lugol’s iodine (12 ml; 100 mg/mL potassium iodide; and 50 mg/mL iodine).

#### TUNEL

Serial sections of 4–5 μm thickness were cut in transverse plane, deparaffinised, and rehydrated in xylene (2 × 5 min), 100%, 95%, 70% ethanol (5 min), and water. Endogenous peroxidase activity was blocked in 3% H2O2 in 1x PBS for 15 min. Sections were washed in water for 5 min. Enzyme digestion was performed with 10 μg/ml Proteinase K in 1x PBS, for 30 min at room temperature. Tissue sections were bordered with a Pap pen, and washed in 1 x PBS-T for 2 × 3 min. Serum-free protein blocking solution was applied, 2–3 drops per slide for 20 min at room temperature (Dako). TdT enzyme was prepared according to the instructions provided by the manufacturer (Terminal Transferase, Biotin-16-dUTP; Roche), and sections were incubated for 1 h at 37 °C. Sections were washed in 1x PBS-T for 3 ×3 min, incubated in Vectastain ABC kit for 30 min at room temperature (Vector Laboratories), washed in 1 x PBS-T for 3 ×3 min, incubated in DAB substrate kit for 10 min (Abcam), washed in water for 5 min, dehydrated through a graded alcohol series, cleared in xylene, and mounted on coverslips.

#### Picro-Sirius Red

Celestine Blue (Celestine Blue 2.5 g, Iron Alum (Ferric ammonium sulfate) 25 g, Distilled water 500 ml). Picro-Sirius Red (0.1% aqueous Sirius Red F3B (CI 35780) 5 ml, Saturated aqueous picric acid 45 ml). Serial sections of 5 μm thickness were cut in transverse plane, deparaffinized in xylene (3 changes, total of 3 min), dipped in 100% ethanol (3 changes, 10 dips each), transferred to water, stained in celestine blue for 5 min, washed in water, stained in Harris' haematoxylin for 5 min, washed in water, differentiated in acid alcohol (5 dips), washed in water, and stained with Pico-Sirius Red for 30 min. After blot drying, sections were dehydrated in 100% ethanol (4x), xylene (3x, 10 dips each), and were coverslipped and mounted in permount. Results: Collagen, reticulin, basement membrane – red. With polarizing microscopy: Large fibers – yellow or orange birefringence; thin fibers – green birefringence; collagen type 4 – not birefringent; nuclei – black; elastin, muscle, cytoplasm – yellow.

### Slide scanning and analysis

Slide scanning was performed at the Imaging Facility at The Hospital for Sick Children, Toronto, Canada (Zeiss Epifluorescence Microscope, 20x/0.75 objective). Elastic trichrome and TUNEL stains: Image visualization and analysis were performed with CaseViewer software (3DHISTECH Ltd.). The thickness of the endo-/subendocardial layer (distance between endothelium and muscle layer in µm) was determined in Elastic trichrome stained sections of left heart free walls. This layer contained endothelium, endocardial connective tissue, subendocardium, and Purkinje fibers. Multiple measurements of thickness were made in each sample (~every 50–100 µm), and a repeated measures analysis was performed as a linear mixed effects model, with treatment as a fixed effect and the sample as a random effect. Picro-Sirius Red stains: Image analysis was performed with Halo software (v3.1.1076.301). Images were analyzed using an adapted version of the Halo Quantification algorithm.

### Bulk RNA sequencing

RNA from the free walls of the LV and RV, AAo, and PA was extracted using the miRNeasy Mini Kit (QIAGEN). Three samples originated from severely hypoplastic hearts (which were also used for single nucleus RNA sequencing), and one came from a moderately hypoplastic heart. Four samples were sequenced as controls. RNA samples had A260/A280 ratios of 2.06–2.07, and were checked for integrity on an Agilent Bioanalyzer 2100 RNA Nano chip. Concentration was measured by Qubit RNA HS Assay on a Qubit fluorometer (Thermo Fisher Scientific, USA). Samples were submitted for small RNA and rRNA-depletion library preparation and sequencing at The Centre for Applied Genomics (TCAG), The Hospital for Sick Children.

For the sequencing of long non-coding RNA and poly(A) mRNA, RNA-Seq library preparation was performed following the TruSeq Stranded Total RNA Library Preparation protocol (Illumina, USA) with the Ribo-Zero Gold rRNA removal kit. Briefly, 600 ng of total RNA was used as the input material and submitted to rRNA depletion using biotinylated beads that contain rRNA-targeting-specific oligos. rRNA-depleted RNA samples were fragmented into ranges of 200–300-bases for 4 min at 94 °C and converted to double-stranded cDNA, end-repaired, and adenylated at the 3’ to create an overhang A to allow for ligation of Illumina adapters with an overhang T. Library fragments were amplified under the following conditions: initial denaturation at 98 °C for 10 s, followed by 12 cycles of 98 °C for 10 s, 60 °C for 30 s, and 72 °C for 30 s, and finally an extension step for 5 min at 72 °C. At the amplification step, each sample was amplified with a different barcoded adapter to allow for multiplex sequencing. One µl of each of the RNA-seq libraries was loaded on a Bioanalyzer 2100 DNA High Sensitivity chip (Agilent Technologies, USA) to check for size and absence of primer dimers; RNA libraries were quantified by qPCR using the Kapa Library Quantification Illumina/ABI Prism Kit protocol (Sigma Aldrich, USA). Libraries were pooled in equimolar quantities and paired-end sequenced on a NovaSeq 6000 platform (Illumina), using 2 lanes of a S2 flowcell following Illumina’s recommended protocol to generate paired-end reads of 150-bases in length, resulting in ~90 M paired-end reads per sample.

Small RNA library preparation was performed following the NEBNext Small RNA Library Prep Set for Illumina protocol (for one aortic sample, library preparation failed). Briefly, 600 ng of total RNA was used to ligate 3′ end Illumina-compatible adaptors, followed by hybridization with the reverse transcription primer that prevents formation of adaptor dimers; then 5’ end adaptors were ligated to the small RNA, and adapter-ligated small RNA molecules were reverse transcribed and amplified by PCR with an initial denaturation at 94 °C for 30 s, followed by 15 cycles of 94 °C for 15 s, 62 °C for 30 s and 70 °C for 15 s, and a final extension step for 5 min at 70 °C. At the amplification step, each sample was amplified with a different barcoded adapter to allow for multiplex sequencing. One µl of each small RNA libraries was loaded on a Bioanalyzer 2100 DNA High Sensitivity chip to check for size and absence of primer dimers. RNA libraries were quantified by qPCR using the Kapa Library Quantification Illumina/ABI Prism Kit protocol. For one aortic sample, library preparation failed twice. Libraries were pooled in equimolar quantities and single-end sequenced for 50-bases on 2 lanes of a Rapid Run Mode flowcell with the V3 sequencing chemistry on a HiSeq 2500 platform (Illumina), following Illumina’s recommended protocol), resulting in almost 8 M reads per sample.

### Bulk RNA sequencing analysis

#### Long non-coding RNA and poly(A) mRNA

Sequencing reads processing: We used STAR and the most updated sheep genome “oviAri4” to map the reads. Desirable mapping rates (85%–90%) were achieved for all the samples and around 80% of mapped reads were successfully assigned to annotated gene bodies, suggesting the overall data quality is high and reliable. Two AAo control samples were removed from the analyses (342284_2, 342287_1), as they were found to express high-levels of myocardial genes (such as, NPPA, MYL4, ANKRD1, MYL7, MYPN, and CSRP3), indicating a potential contamination with cardiac tissue. Exons were counted using featureCounts to the Oar_V4.0 genome annotation. The correlation of gene expression values between all samples was used to assess the quality of the dataset.

Differential expression analysis: Counts were normalized with RUV-seq^60^ and differentially expressed genes were computed using edgeR (log2 fold change > 1 and false discovery rate (FDR)-adjusted p-value < 0.05). Briefly, small RNA-seq reads had adapter sequences trimmed with the BBMap suite to keep reads with fewer than 23 nucleotides after trimming. Reads were aligned to the OviAri4 genome and Oar_v4.0 annotation using miRDeep2.

#### Small RNA

Sequencing reads processing: FastQC (v0.11.7) was used to examine the quality of sequenced reads (https://www.bioinformatics.babraham.ac.uk/projects/fastqc/). BBDuk (BBMap suite v37.90) (https://sourceforge.net/projects/bbmap/) was used to trim adapter sequences from reads with reference adapter sequences provided by BBMap suite and settings “hdist=1 mink=11” for small RNA-seq reads. For miRNA size specificity, only reads <23 nucleotides in length were retained. Following trimming, FastQC was used to examine the quality of trimmed sequenced reads. miRDeep2 mapper.pl^61^ was used with default parameters to map reads of at least 18 nucleotides in length to the sheep (Ovis aries) genome (Oar_v4.0). Known and novel miRNAs were identified using miRDeep2 main algorithm (miRDeep2.pl) with default parameters. For known miRNAs, the mature miRNA sequences in sheep were obtained from miRBase (v22.1)^62^. For novel miRNAs, only those with miRDeep score ≥2 were retained for downstream analysis.

Differential miRNA expression analysis: Differential miRNA expression analysis was performed in R (v4.0.3). First, novel miRNAs that appear in <75% of replicates within a given tissue-treatment group were excluded. Second, novel and mature miRNAs with log-transformed counts ≥1 in 75% of replicates for a given tissue-treatment sample were retained for downstream analysis. RUV-seq (v1.24.0)^60^ was applied to remove unwanted variation using replicate samples (RUVs with k = 2). Differentially expressed (DE) miRNAs were identified (absolute log2FC > 1, FDR-adjusted p-value < 0.05; Supplementary Data 1) for treatment – control within each tissue using DESeq2 (v 1.30.1)^63^. Shrunken log2FCs were calculated with “normal” shrinkage estimator. Mature sequences of DE novel miRNAs were used to identify known miRNA homologs based on sequence similarity in other species with “Single sequence search” function on miRBase (v22.1) with SSEARCH search method.

miRNA-target gene identification: Computationally predicted and experimentally validated target genes of differentially expressed miRNAs in AAo and LV were identified using multiMiR (v1.12.0)^64^. For experimentally validated target genes, sources were limited to “miRTarBase”, “TarBase” and “miRecords” using the “validated” parameter in multiMiR. Human homologs of DE miRNAs were identified from mature miRNA sequence with SSEARCH search method on miRBase (v22.1) and used as input. If no human homologs could be identified, then the DE miRNA was excluded from gene-target identification. Potential target genes were pre-filtered for differentially expressed genes in AAo or LV with opposing expression differences between coil-treated and control compared to the differentially expressed miRNA in the same respective tissue. For example, target genes of a miRNA more highly expressed in coil-treated AAo samples would be identified from genes more highly expressed in control AAo samples. Identified target genes were then converted from human to sheep orthologs using biomaRt (v2.46.3). Missing orthologs were manually retrieved using NCBI’s Gene resource.

### Single-nucleus RNA sequencing

Single-nucleus RNA sequencing (snRNA-seq) was performed at the Princess Margaret Genomics Centre, Toronto, Canada. The experimental design consisted of four lamb hypoplastic left heart samples and three controls. Tissue of the left ventricular free wall (30–50 mg per sample) was mechanically dissociated into 1–2 mm^3^ using chilled razor blades (Fisher Scientific). In order to isolate nuclei, the tissue was suspended in lysis buffer (for 5 min) and homogenized using a Dounce homogenizer (Sigma-Aldrich). Intact nuclei were verified by SYBR Green II RNA Gel Stain (10,000X concentrate in DMSO; Thermo Fisher Scientific). Resuspended nuclei were counted and pellets were washed twice in resuspension buffer. The resuspension was filtered with a 40 µm Flowmi cell strainer (Sigma-Aldrich), and transferred to a 1.5 ml LoBind tube (Sigma-Aldrich). Nuclei were counted again and incubated with DAPI (Sigma-Aldrich), at concentrations suggested by the manufacturer. To exclude debris or nuclei aggregates, fluorescence-activated cell sorting (FACS) was performed with a BD Influx cell sorter (BD Biosciences), gating for DAPI positivity (for 1–1.5 h). Nuclei were collected and washed with resuspension buffer, and then counted. 10x Chromium single-cell gene expression technology (3′ v2 10x Genomics) was used to generate single-indexed libraries as per the manufacturer’s protocols. RNA sequencing was performed on a HiSeq X (LV 1 and 2) or NovaSeq 6000 platform (LV 3 and 4) (Illumina), as per the manufacturer’s recommendations.

#### Lysis buffer

0.32 mM sucrose (Sigma-Aldrich), 5 mM CaCl_2_ (Sigma-Aldrich), 3 mM Mg(Ac)_2_ (Sigma-Aldrich), 20 mM Tris-HCl 7.5 (Fisher Scientific), 0.1% Triton X-100 (Sigma-Aldrich), 0.1 mM EDTA 8.0 (Sigma-Aldrich), 40 U/ml RNase Inhibitor (Sigma-Aldrich), in UltraPure DNase/RNase-Free Distilled H_2_O (Fisher Scientific). Resuspension buffer: 1x PBS (pH 7.4; Thermo Fisher Scientific), 1% BSA (MACS Miltenyi Biotec), and 0.2 U/µl RNase Inhibitor (Sigma-Aldrich).

### De novo annotations of Ovis aries reference transcriptome

An initial mapping of snRNA-seq reads was performed using Cell Ranger v3 (10x Genomics) and a ‘pre-mrna’ (i.e., pre-splicing) reference transcriptome, derived from the *O. aries* NCBI genome assembly 4.0. This approach resulted in ~25% sequencing reads mapping to putative intergenic regions. For comparison, human snRNA-seq samples typically produce <3% of reads mapping to intergenic regions. We hypothesized that some of the *O. aries* putative intergenic regions were in fact unannotated open reading frames. To test this, we pooled bulk RNA sequencing measurements from LV, AAo, RV, and PA for a de novo annotation of the *O. aries* transcriptome, following the STAR alternate protocols 3 and 8^65^, with the NCBI genome assembly 4.0 as a template. This reduced the proportion of snRNA-seq reads mapping to putative intergenic regions (i.e., those not measured by bulk RNA sequencing) to ~2.5% (Supplementary Data 4). For further analyses, we used de novo transcriptome annotations, including extensions of the NCBI genome assembly 4.0 gene coordinates and new open reading frames (coordinates provided in Supplementary Data 5).

### SnRNA-seq bioinformatic analyses

Single nucleus sequencing reads were mapped to the de novo reference transcriptome using Cell Ranger v3 (10x Genomics). All samples passed bioinformatics quality control (Supplementary Data 4). We measured expression of 50–8000 genes per nucleus, and less than 30,000 unique molecular identifiers, which are both indicators of good quality data (e.g., not suggesting a major contribution of droplet doublets). As expected for single nucleus data, no mitochondrial RNA was detected. The raw gene × nucleus read counts were normalized using SCTransform^66^, and all seven samples were integrated with Seurat’s integration anchors, using Canonical Correlation Analysis, and clustered using Seurat^67^, as implemented in CReSCENT multi-sample pipeline^68^. This pipeline included batch effect correction, data dimension reduction, cell clustering (Supplementary Data 6), and visualization using the Uniform Manifold Approximation and Projection (UMAP)^69^. Cell identities were assigned to each cluster, comparing average gene expression profiles for each cluster against manually curated cardiac cell type signatures (Supplementary Data 7), using Gene Set Variation Analysis (GSVA; Supplementary Data 8)^70^ as previously described^71^. The cell identities were cross-referenced with known markers. Nuclei from cluster 7 were re-clustered and relabeled, using the same cell type signatures and GSVA.

### Cell type composition analyses

We analyzed the differential abundance of nuclei in coiled versus control samples by two methods. First, we pooled the nucleic numbers in 3 coiled samples and 3 controls (one coiled sample was excluded because of much higher number of adipose tissue nuclei compared to the others; see below), and performed a Fisher’s exact test (FET) of the fraction of each cell type. Second, we performed multinomial logistic regression analyses using *scCODA*^36^ and *Dirichlet Reg*^37,38^. The first method *scCODA* is available from the Theis lab (https://github.com/theislab/scCODA)^36^. *ScCoda* is a Bayesian method and uses a continuous logit-normal variant of the slab-and-spike prior^72^ as a method of variable (cell type) selection, that identifies only large contributors to cellular composition change. The second method *DirichletReg* (https://CRAN.R-project.org/package=DirichletReg, https://epub.wu.ac.at/4077/) has previously been used to analyze cell composition in intestinal biopsies from human ulcerative colitis^37^ and has also been compared to the *scCODA* package, both in the preprint and GitHub site. Our initial focus was on 4 cell types: cardiomyocytes, fibroblasts, endothelial cells, and adipocytes. Sample LV_2_coiled was atypical with the smallest number of total nuclei, largest number of adipocytes, and fewest cardiomyocytes. We have adopted a conservative approach and treated this sample as a likely outlier representing a region with a deposit of adipose tissue, and excluded it from inferences on changes in cell composition that could be attributed to changes secondary to low left heart flow.

### SnRNA-seq trajectory and differential expression analyses

We separated snRNA-seq data from all samples into three manifolds. The cardiomyocyte manifold contained clusters 10, 3, and 0. The fibroblast manifold contained clusters 2 and 1. The endothelial manifold contained clusters 15 and 5. We then further filtered nuclei in the cardiomyocyte manifold that were not within the manifold. We inspected the UMAP with the “DimPlot” function in Seurat V4^67,73^ and filtered cells with UMAP_1 > 5 and UMAP_2 < 6. We measured pseudotime trajectories with Slingshot^74^, with stretch = 0 and extension = “n”. We used clusters 10, 2, and 15 as the start clusters for the cardiomyocyte, fibroblast, and endothelial clusters, respectively. First, we removed genes that were not designated with a gene symbol. We then used the tradeSeq^75^ package to estimate the minimum number of appropriate knots with the “evaluateK”^75^ function before fitting a negative-binomial general additive model (nb-GAM) for each gene with the “fitGAM”^75^ function. The 5,000 most variable genes also had nb-GAM’s fitted in each condition to allow for differences in expression between conditions along pseudotime. We identified genes whose expression is associated with pseudotime using the “associationTest” function^75^, and genes that were differentially expressed between conditions using the “conditionTest”. We corrected p-values returned with these functions using a false-discovery rate (cut-off: FDR-adjusted *p*-value < 0.05). We then generated pseudotime heatmaps of associated and differentially expressed genes by predicting smoothers for each gene using the “predictSmooth”^75^ function. Smoothers were scaled and then plotted with the pheatmap function. We completed pathway enrichment of these associated and differentially expressed genes using the following command:^76^ gprofiler(genes,”hsapiens”, ordered = TRUE, src_filter = c(“GO:BP”, “REAC”, “KEGG”), custom_bg = detected_genes, correction_method = “fdr”)^77^ and plotted with ggplot2.

Cluster-specific differentially expressed genes were measured between coiled and control samples, using the MAST method, “Find Markers” function within Seurat V4 package. Genes were considered differentially expressed if the gene was detected in more than 10% of the cells within either cluster and if the gene had an FDR-adjusted p-value < 0.05. Pathway enrichment of differentially expressed genes was completed with the gProfileR R package, using the following command: gprofiler(genes,”hsapiens”,ordered = TRUE,src_filter = c(“GO:BP”,”REAC”,”KEGG”),custom_bg = detected genes,correction_method =”fdr”).

### Cellular signaling analyses

We performed cell-signaling analysis using the CellChat R package by following their published workflows (https://github.com/sqjin/CellChat/tree/master/tutorial)^78^. Specifically, we split the Seurat object into the two conditions “controls” and “coiled”, before extracting the necessary data for CellChat, following the “Interface with other single-cell analysis toolkits” tutorial. Briefly, each dataset was converted into a CellChat object. Cellular communication was estimated with the “computeCommunProb” and “filterCommunication” functions against the “CellChatDB.human” ligand-receptor database. Ligand-receptor pairs were organized into signaling pathways using the “computeCommunProbPathway” function. Cellchat objects were merged before shared and condition-specific cellular communication was computed with a joint manifold learning and toplogical similarity approach using the “computeNetSimilarityPairwise”, “netEmbedding”, and “netClustering” functions. Conserved and condition-specific signaling pathways were then visualized with the “netAnalysis_signalingRole_heatmap” function. Differentially regulated ligand-receptor pairs computed with the “identifyOverExpressedGenes” and “netMappingDEG” functions in CellChat.

### Statistics and reproducibility

All data processing for bulk RNA-seq, small RNA-seq, and snRNA-seq were performed with open-source statistical packages using default parameters unless otherwise specified in the Materials and Methods. After data processing, statistical analyses were performed in R-/4.0.0.

### Reporting summary

Further information on research design is available in the Nature Portfolio Reporting Summary linked to this article.

## Supplementary information

Supplemental Material

Description of Additional Supplementary Files

Supplementary Data 1

Supplementary Data 2

Supplementary Data 3

Supplementary Data 4-8

Reporting Summary

## Data Availability

Sequence data is available through ArrayExpress (accession numbers: E-MTAB-12327 and E-MTAB-12230) and the Broad Institute (SCP1994). Additional data is available through 10.6084/m9.figshare.23511888.
