# Supplementary material for: Decreased left heart flow in fetal lambs causes left heart hypoplasia and pro-fibrotic tissue remodeling
Source: Commun Biol. 2023 Jul 22;6:770. doi: 10.1038/s42003-023-05132-2 (PMC10363152; doi:10.1038/s42003-023-05132-2)
Supplement: Supplementary file 1 — Supplemental Material [file 42003_2023_5132_MOESM1_ESM.pdf]

Supplementary material for:

Decreased left heart flow in fetal lambs causes left heart hypoplasia and pro-fibrotic tissue remodeling

Miriam S. Reuter, Dustin J. Sokolowski, Javier Diaz-Mejia, Johannes Keunen, Barbra de Vrijer, Cadia Chan, Liangxi Wang, Greg Ryan, David A. Chiasson, Troy Ketela, Stephen W. Scherer, Michael D. Wilson, Edgar Jaeggi, Rajiv R. Chaturvedi

Table S1. Cell composition of the myocardium, as reported from different studies, methodologies, species, developmental stages, and tissue types.

Study	Methodology	Species	Age	Tissue	CM	FB	EC	SMC	Other
Pinto et al. [1]	Immunohistochemistry	Mouse	Adult	LV	33%	13%	55%	6%	9% leukocytes
		Human	Adult	LV	31%	NA	54%	NA	3% leukocytes
Bergmann et al. [2]	Stereology	Human	Postnatal	LV	66%				
			Young adults	LV	18%		24%		58% mesenchymal cells
	Flow cytometry	Human	Young adults	LV	33%		24%		43% mesenchymal cells
Banerjee et al. [3]	Flow cytometry	Mouse	E18.5	Whole heart	65%	14%			
			P1	Whole heart	60%	10%			
			P5	Whole heart	76%	14%			
			P15	Whole heart	63%	18%			
			Adult	Whole heart	56%	27%	7%	10%	
		Rat	P1	Whole heart	62%	30%			
			P15	Whole heart	30%	64%			
Cui et al. [4]	Single-cell RNA sequencing (n = 3,842 cells)	Human	Embryonic and fetal	Various regions	43%	17%	16%		
DeLaughter et al. [5]	Single-cell RNA sequencing (n = 70 cells)	Mouse	E11.5	LV	86%	0%	14%		
	Single-cell RNA sequencing (n = 138 cells)		E14.5	LV	63%	22%	15%		
	Single-cell RNA sequencing (n = 53 cells)		E18.5	LV	51%	26%	23%		
	Single-cell RNA sequencing (n = 124 cells)		P0	LV	59%	26%	15%		
	Single-cell RNA sequencing (n = 60 cells)		P3	LV	18%	62%	20%		
	Single-cell RNA sequencing (n = 73 cells)		P21	LV	88%	8%	4%		
Li et al. [6]	Single-cell RNA sequencing (n = 55 cells)	Mouse	E8.5	LV/RV	76%				

	Single-cell RNA sequencing (n = 118 cells)		E9.5	LV	72%		14%		5% mesenchymal cells
	Single-cell RNA sequencing (n = 115 cells)		E10.5	LV	53%		20%		13% mesenchymal cells
Xiao et al. [7]	Single-cell RNA sequencing (n = 4,406 cells)	Mouse	E13.5	Whole heart	37%	6%	21%	7%	
	Single-cell RNA sequencing (n = 3,920 cells)		E14.5	Whole heart	52%	8%	18%	3%	
Hu et al. [8]	Single-nucleus RNA sequencing (n = 7,760 control nuclei)	Mouse	P10	LV/RV	59%	19%			
Litvinukova et al. [9]	Single-nucleus RNA sequencing (n = 363,213 nuclei from six anatomical regions)	Human	Adult	LV	51%	14%	8%	2%	5% immune cells
Tucker et al. [10]	Single-nucleus RNA sequencing (n = 287,269 nuclei)	Human	Adult	Four chambers	36%	32%			
Wolfien et al. [11]	Single-nucleus RNA sequencing (n = 8,635 nuclei)	Human	Adult	Whole heart	23%	25%	30%		
This study	Single-nucleus RNA sequencing (n = 12,992 control nuclei)	Lamb	0.84 gestation	LV					

Abbreviations: CM, cardiomyocytes; EC, endothelial cells; FB, fibroblasts, SMC, smooth muscle cells; LV, left ventricle; RV, right ventricle.

Table S2. Fetal assessments. (a) Gestational age/fraction at coil implantation and harvest. (b) Fetal sex and cardiovascular measurements (controls vs. coiled with retrograde AAo flow; and controls vs. coiled with no retrograde AAo flow (sham controls)), at 0.84 gestation.

a

	**Days**		**Fraction of gestation**	
	Median (IQR)	Mean ± SD	Median (IQR)	Mean ± SD
**Implant**	76 (75, 80)	77 ± 2.36	0.52 (0.51, 0.54)	0.52 ± 0.02
**Harvest**	124 (123, 125)	123.1 ± 2.6	0.84 (0.84, 0.85)	0.84 ± 0.02
**Harvest-implant interval**	46 (45, 48)	46.1 ± 2.47	0.31 (0.31, 0.33)	0.31 ± 0.02

b

	**Controls (n = 12)**<br>Mean ± SD	**Retrograde AAo flow (n = 9)**<br>Mean ± SD	***p* value**
**Fetal sex**	Female: 7<br>Male: 3<br>ND: 2	Female: 3<br>Male: 4<br>ND: 2	
**Fetal body weight**	3.0 ± 0.78 kg	3.28 ± 0.68 kg	0.39
**Aortic / pulmonary valve diameter***	0.87 ± 0.12	0.54 ± 0.11	0.00015
**AAo / pulmonary artery diameter***	0.83 ± 0.14	0.58 ± 0.07	0.00036
**LV / RV end-diastolic diameter***	1.07 ± 0.09	0.49 ± 0.16	0.00002
**LV / RV end-diastolic length***	1.16 ± 0.09	0.82 ± 0.21	0.00041
**LV free wall / total heart weight#**	0.23 ± 0.04	0.14 ± 0.01	0.008

	**Controls (n = 12)**<br>Mean ± SD	**Sham controls (n = 5)**<br>Mean ± SD	***p* value**
**Fetal sex**	Female: 7<br>Male: 3<br>ND: 2	Female: 1<br>Male: 3<br>ND: 1	
**Fetal body weight**	3.0 ± 0.78 kg	4.0 ± 0.78 kg	0.04
**Aortic / pulmonary valve diameter***	0.87 ± 0.12	0.72 ± 0.17	0.09
**AAo / pulmonary artery diameter***	0.83 ± 0.14	0.76 ± 0.08	0.3284
**LV / RV end-diastolic diameter***	1.07 ± 0.09	0.89 ± 0.15	0.09
**LV / RV end-diastolic length***	1.16 ± 0.09	1.0 ± 0.26	0.07
**LV free wall / total heart weight#**	0.23 ± 0.04	0.19 ± 0.08	0.5714

Due to fetal size variations, left heart echocardiographic measurements were normalized to the corresponding right heart structures. The weight of the LV free wall was normalized to the total heart weight. No differences were found between the total fetal body weight of cases vs. controls.

* Echocardiographic measurements.

# Autopsy measurements.

Abbreviations: AAo, ascending aorta; IQR, interquartile range; LV, left ventricle; ND, not documented; RV, right ventricle; SD, standard deviation.

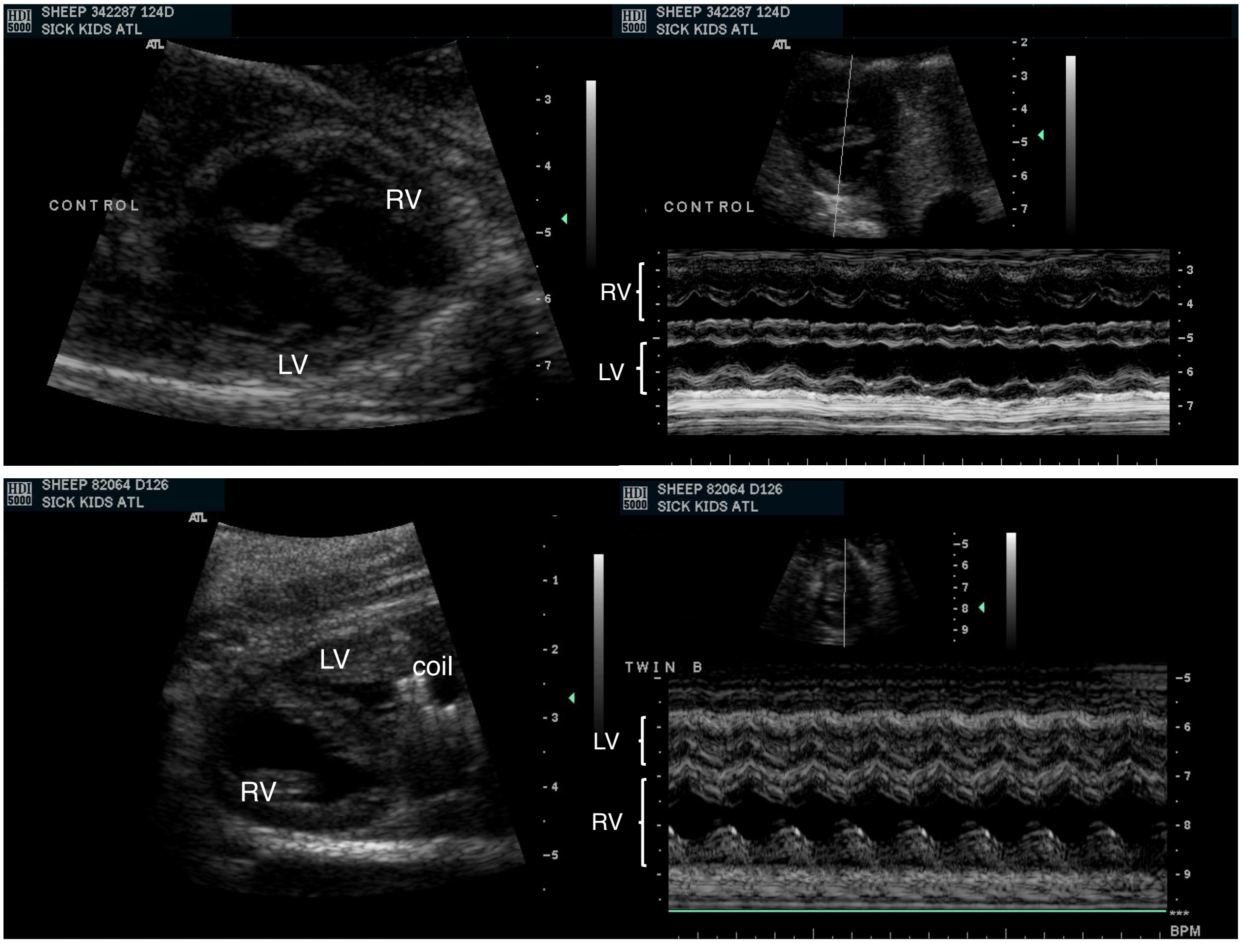

Figure S1. Echocardiographic imaging of a control (upper panel) and a hypoplastic left heart (0.84 gestation; lower panel). Four chamber view (left panel). M-mode (right panel): Longitudinal display of left and right ventricular contractions.

Abbreviations: LV, left ventricle; RV, right ventricle.

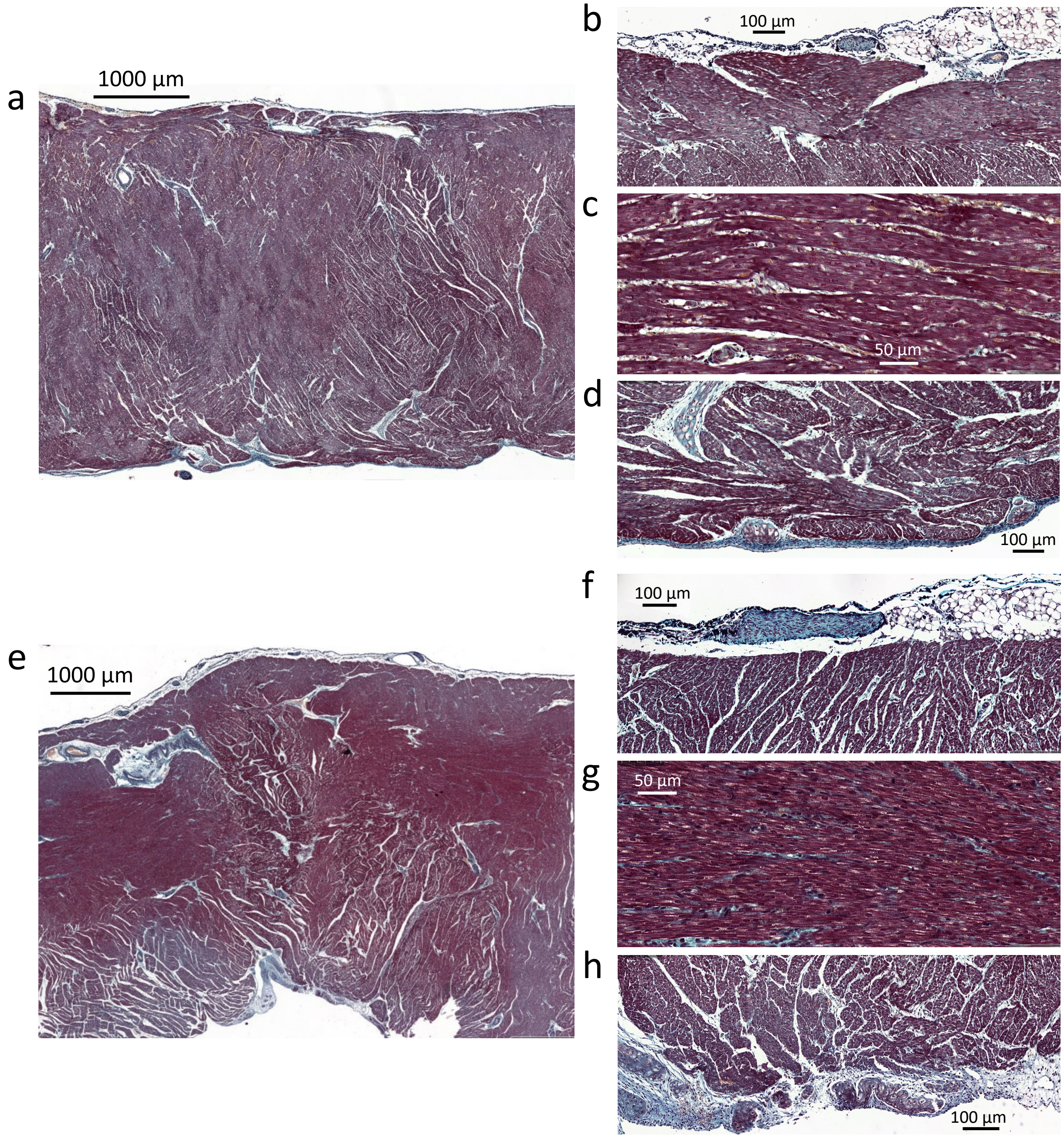

Figure S2. Histology (elastic trichrome stain) of ovine fetal left ventricular free-wall tissue (0.84 gestation). Control: a, b, c, d (same sample at different magnifications and regions). Coiled (severe hypoplasia): e, f, g, h (same sample at different magnifications and regions).

a + e: Overview: Epicardial surface (top), endocardial surface (bottom). b + f: Epicardium and myocardium, with adipocytes. c + g: Myocardium, compact layer. d + h: Myocardium, subendocardium and endocardium.

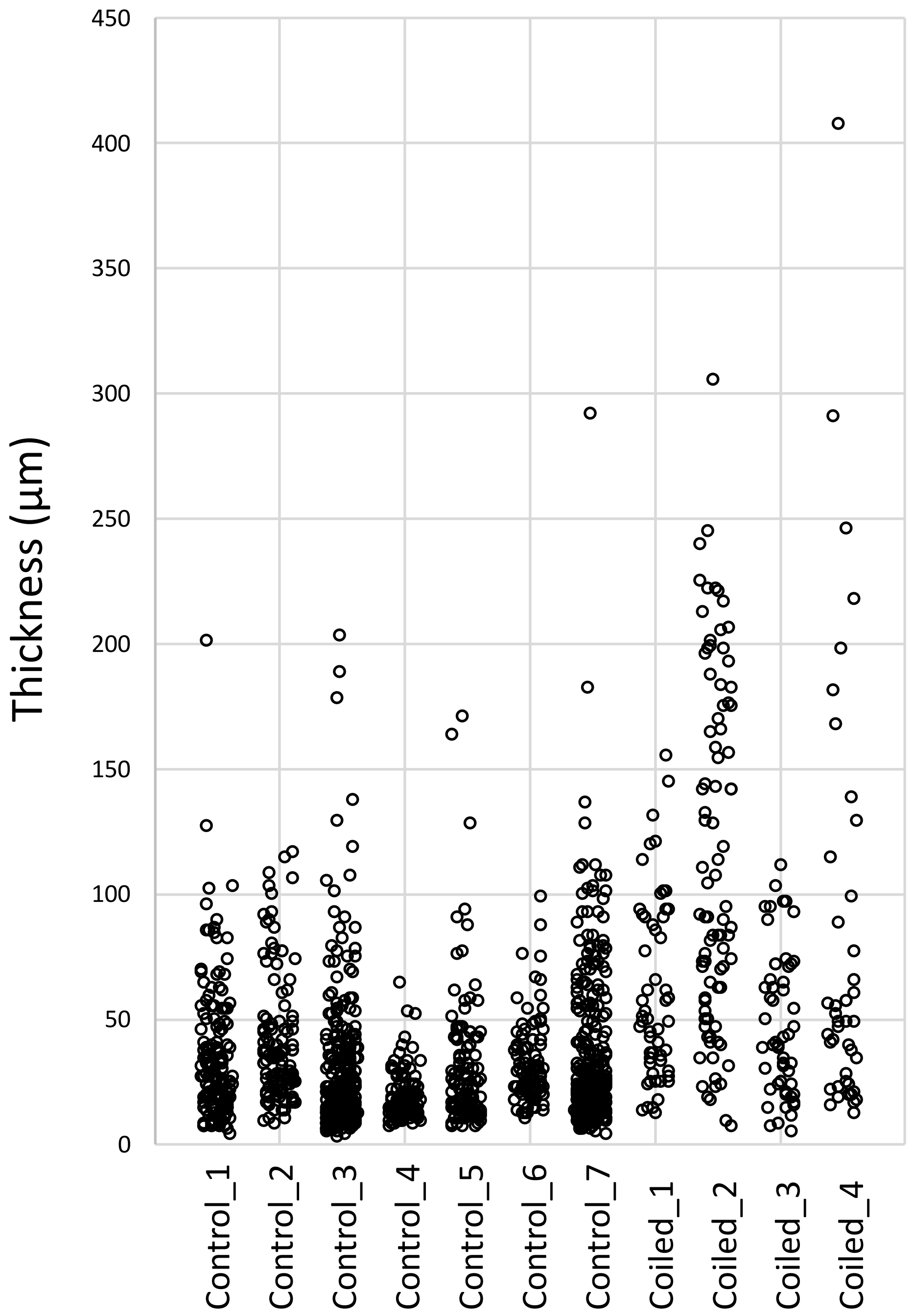

Figure S3. Endo-/subendocardial thickness in controls (n = 7) and severely hypoplastic left ventricular myocardium (n = 4). Each datapoint represents one measurement. Sample size: n = 7 biologically independent controls, n = 4 biologically independent coiled lambs.

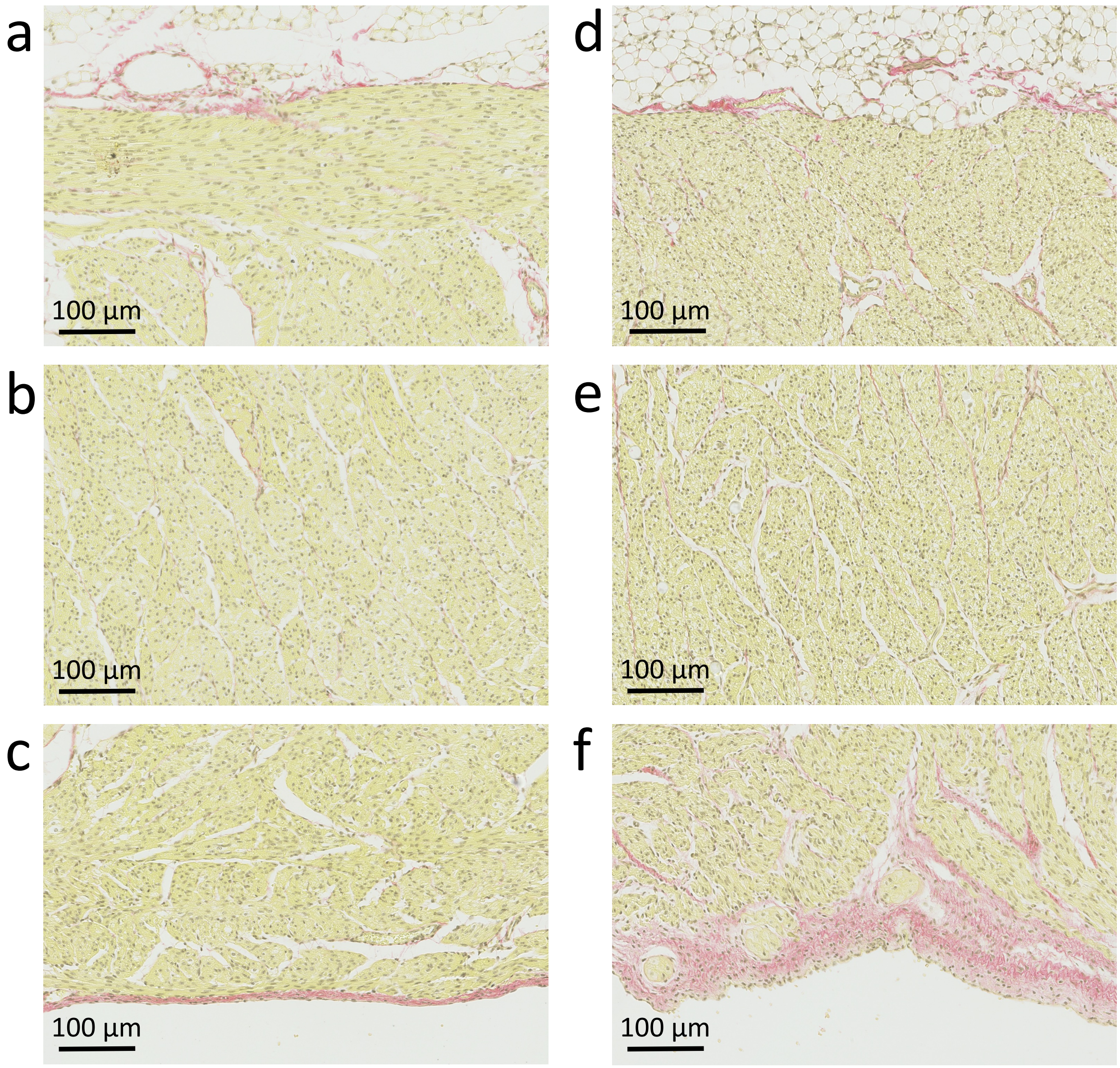

Figure S4. Histology (Picro-Sirius Red staining) of ovine fetal left ventricular free-wall tissue (0.84 gestation). Control: a, b, c. Coiled (severe hypoplasia): d, e, f.

a + d: Epicardium and myocardium. b + e: Myocardium, compact layer. c + f: Myocardium, subendocardium and endocardium.

No statistical difference was found in Picro-Sirius Red staining in n = 4 coiled samples vs. n = 6 controls (controls: 5.32%, coiled: 5.79%; Wilcoxon rank sum test (Mann-Whitney) p = 0.48), however there were various regions with increased subendocardial staining in coiled tissues (f).

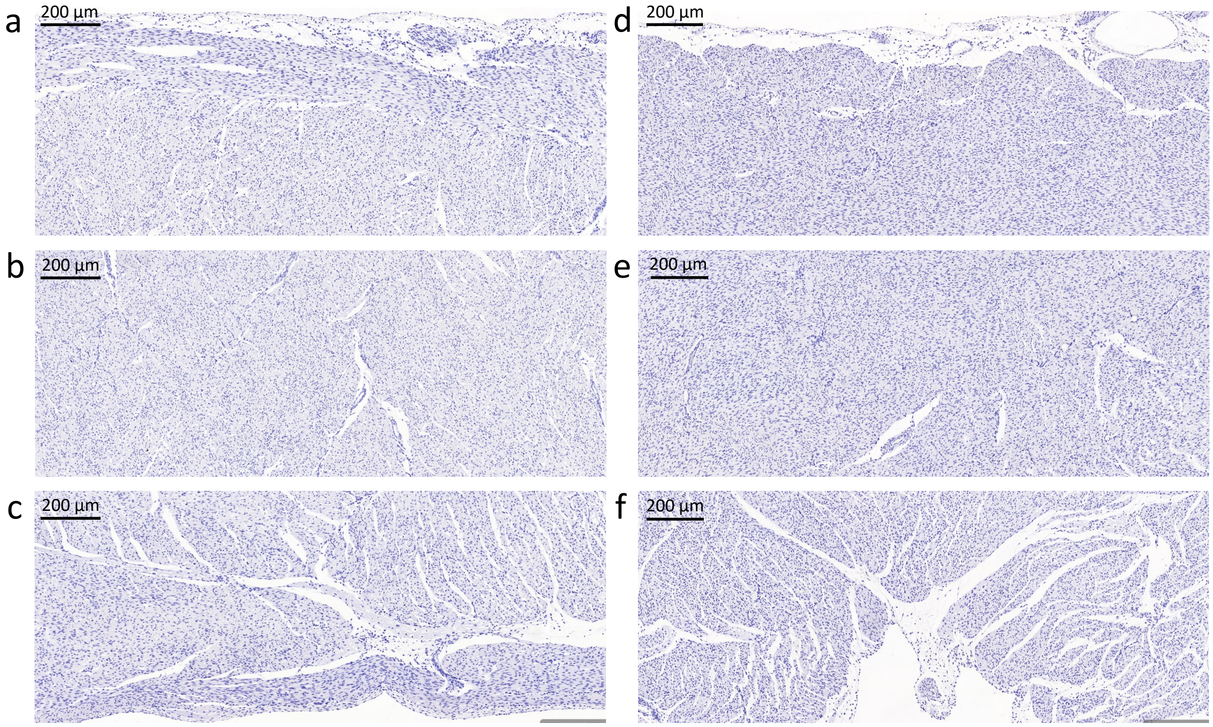

Figure S5. Histology (TUNEL stain) of ovine fetal left ventricular free-wall tissue (0.84 gestation). Control: a, b, c. Coiled (severe hypoplasia): d, e, f.

a + d: Epicardium and myocardium. b + e: Myocardium, compact layer. c + f: Myocardium, subendocardium and endocardium.

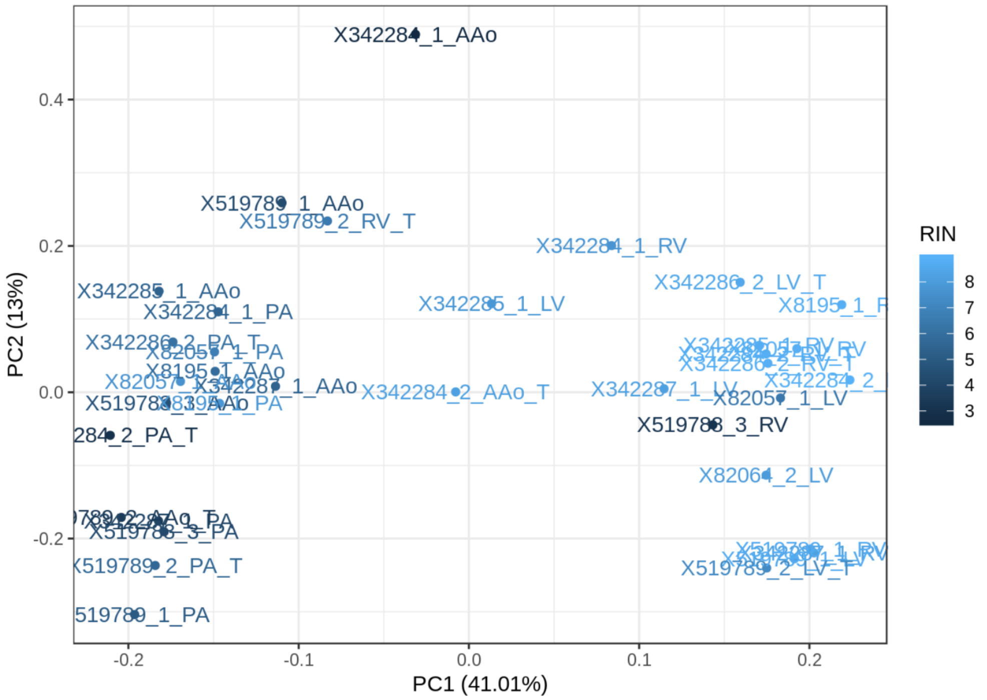

Figure S6. Principle component analysis of bulk RNA sequencing data. Expression profiles clustered by tissue, however there was considerable inter-sample variation.

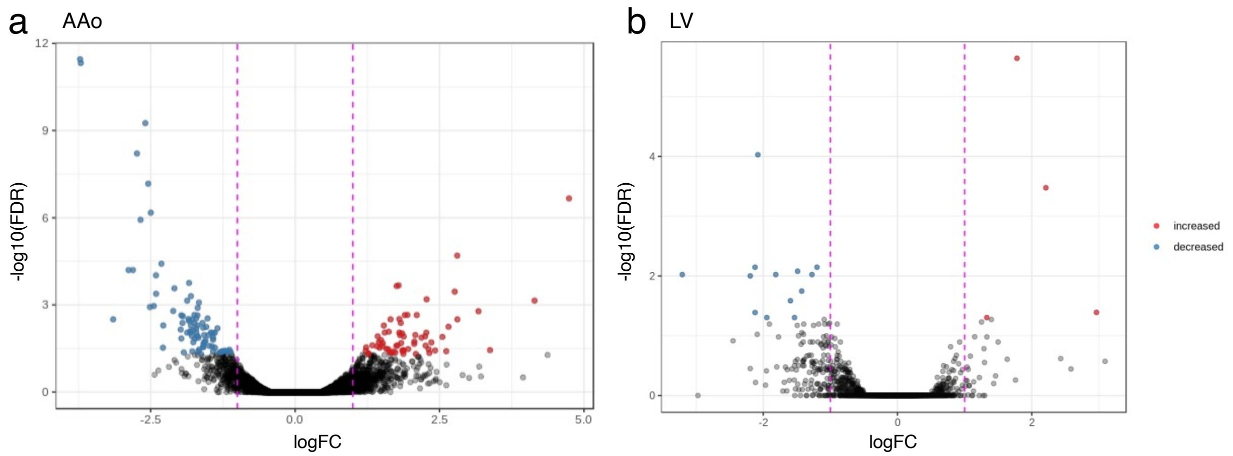

Figure S7. Volcano plot of differentially expressed genes in ascending aorta (AAo; a) and left ventricle (LV; b), from bulk RNA-seq data (coiled versus control tissue). Gene lists are provided in Supplementary Data 1. Sample size AAo: n = 2 biologically independent controls, n = 4 biologically independent coiled lambs. Sample size LV: n = 4 biologically independent controls, n = 4 biologically independent coiled lambs.

a

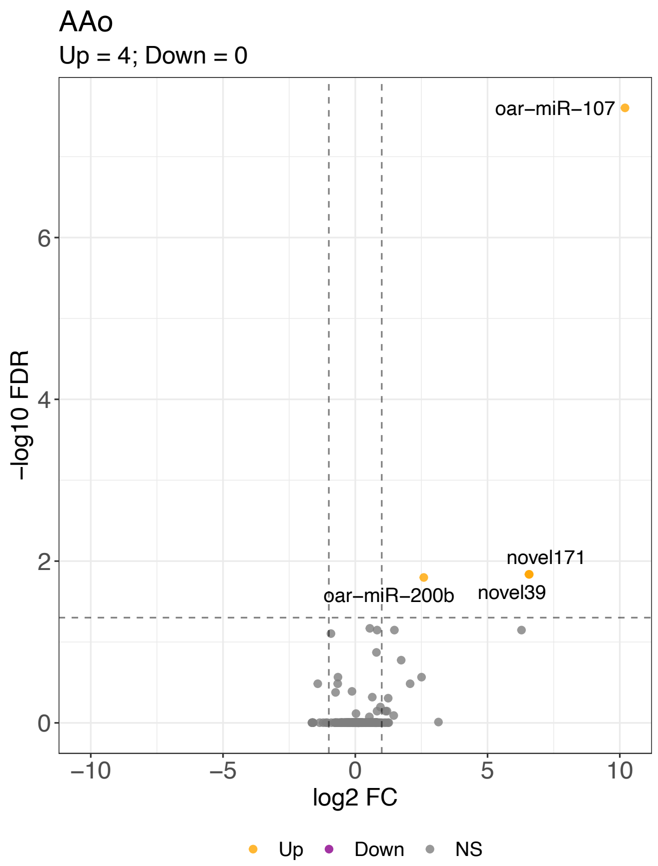

miRNA	mirna_homolog	Species	Direction	log2FC
novel39	miR-363-3p	goat,cow,human,mouse,zebrafish,rat	Up	6.564
novel171	miR-2284x	cow	Up	6.578
oar-miR-200b		sheep	Up	2.589
oar-miR-107		sheep	Up	10.196

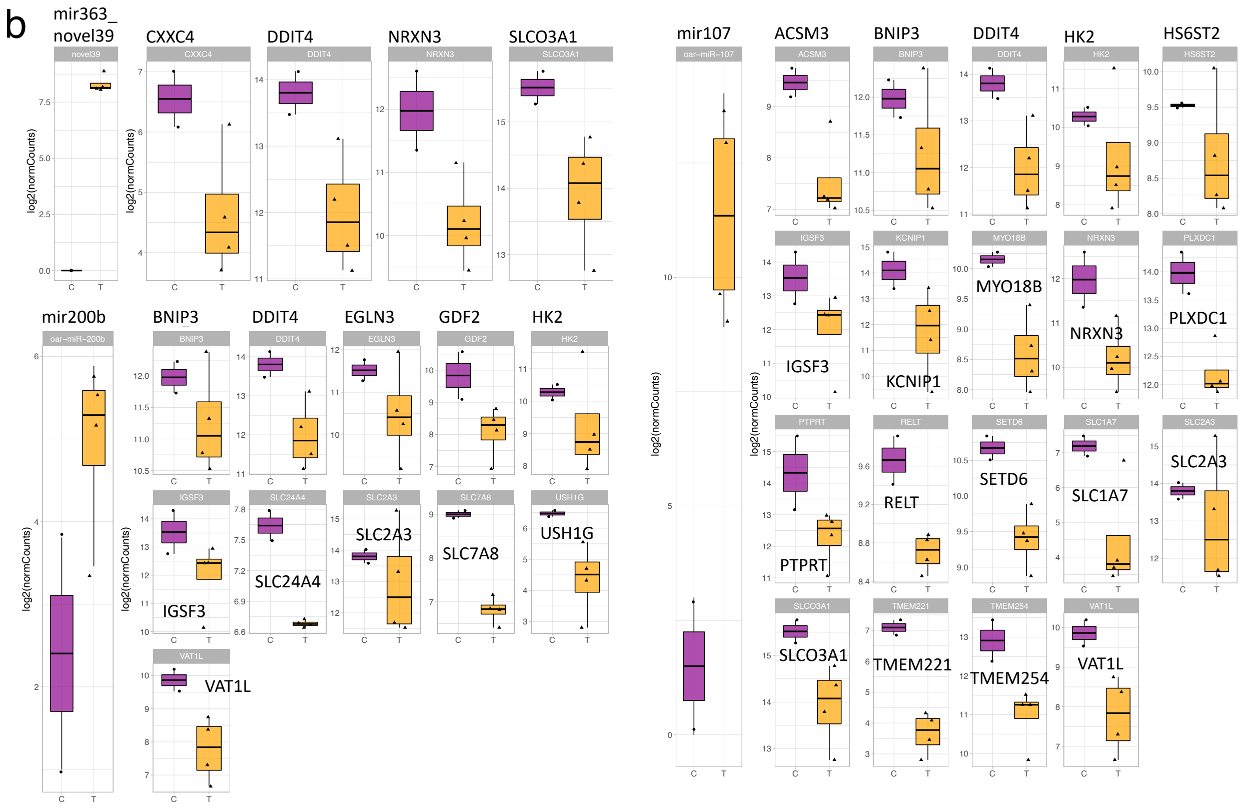

Figure S8. Differentially expressed microRNA (miRNA) in ascending aorta (AAo), from bulk RNA-seq data (coiled versus control tissue). (a) Volcano plot. Scattered points represent miRNA: the x-axis represents the log 2 fold change (log2FC), whereas the y-axis represents the -log10 false discovery rate. Orange dots are miRNA significantly upregulated after coil treatment. The table lists upregulated miRNA (for novel miRNA, homologs from other species were determined based on sequence similarity). (b) Upregulated miRNA and downregulated target genes, coiled (T; treatment) versus control tissue (C). Sample size: n = 2 biologically independent controls, n = 4 biologically independent coiled lambs.

a

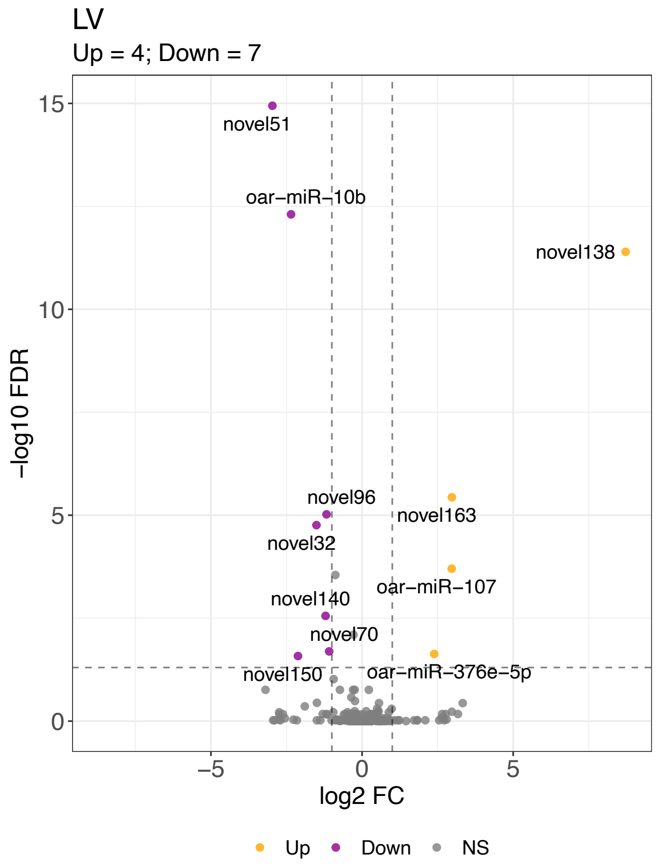

miRNA	mirna_homolog	Species	Direction	log2FC
novel163	miR-125a	cow,pig,chinese treeshrew	Up	2.971
oar-miR-107		sheep	Up	2.967
oar-miR-376e-5p		sheep	Up	2.388
novel138	miR-15a-5p	human,mouse,chicken,cow,pig	Up	8.720
novel32	miR-708-5p	mouse,human,rat,dog	Down	-1.509
novel51	miR-504-5p	cow,rat,human,mouse,goat	Down	-2.968
novel70	miR-425-5p	guinea pig,frog,armadillo,rabbit	Down	-1.089
novel96	miR-106b-3p	mouse,human,rat	Down	-1.176
novel150	miR-664	cow,pig	Down	-2.120
novel140	miR-15b-5p	mouse,human,pig,rat	Down	-1.208
oar-miR-10b		sheep	Down	-2.350

b

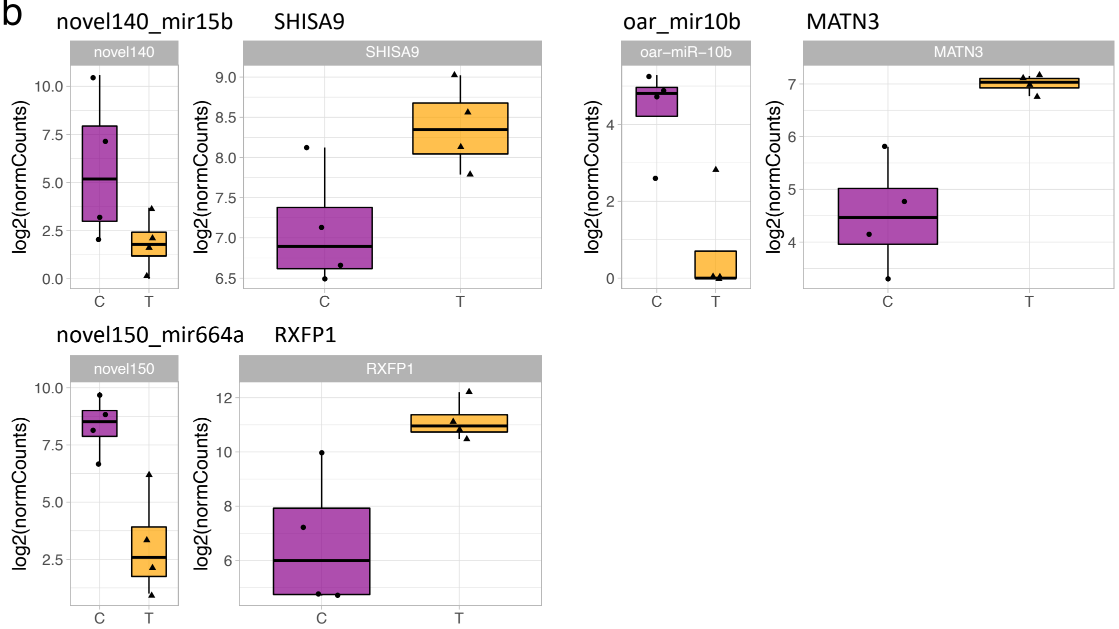

c

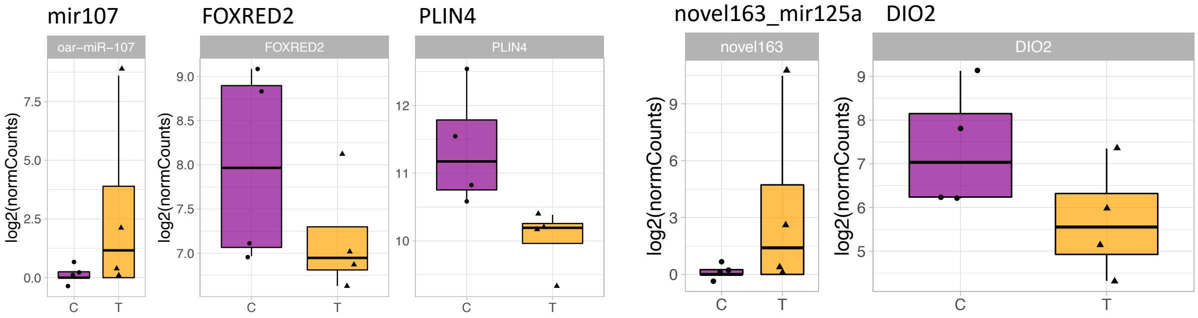

Figure S9. Differentially expressed microRNA (miRNA) in left ventricle (LV), from bulk RNA-seq data (coiled versus control tissue). (a) Volcano plot. Scattered points represent miRNA: the x-axis represents the log 2 fold change (log2FC), whereas the y-axis represents the -log10 false discovery rate. Orange dots are miRNA significantly upregulated after coil treatment, purple dots are significantly downregulated miRNA. The table lists up- and downregulated miRNA (for novel miRNA, homologs from other species were determined based on sequence similarity). (b) Upregulated miRNA and downregulated target genes, coiled (T; treatment) versus control tissue (C). (c) Downregulated miRNA and upregulated target genes. Sample size: n = 4 biologically independent controls, n = 4 biologically independent coiled lambs.

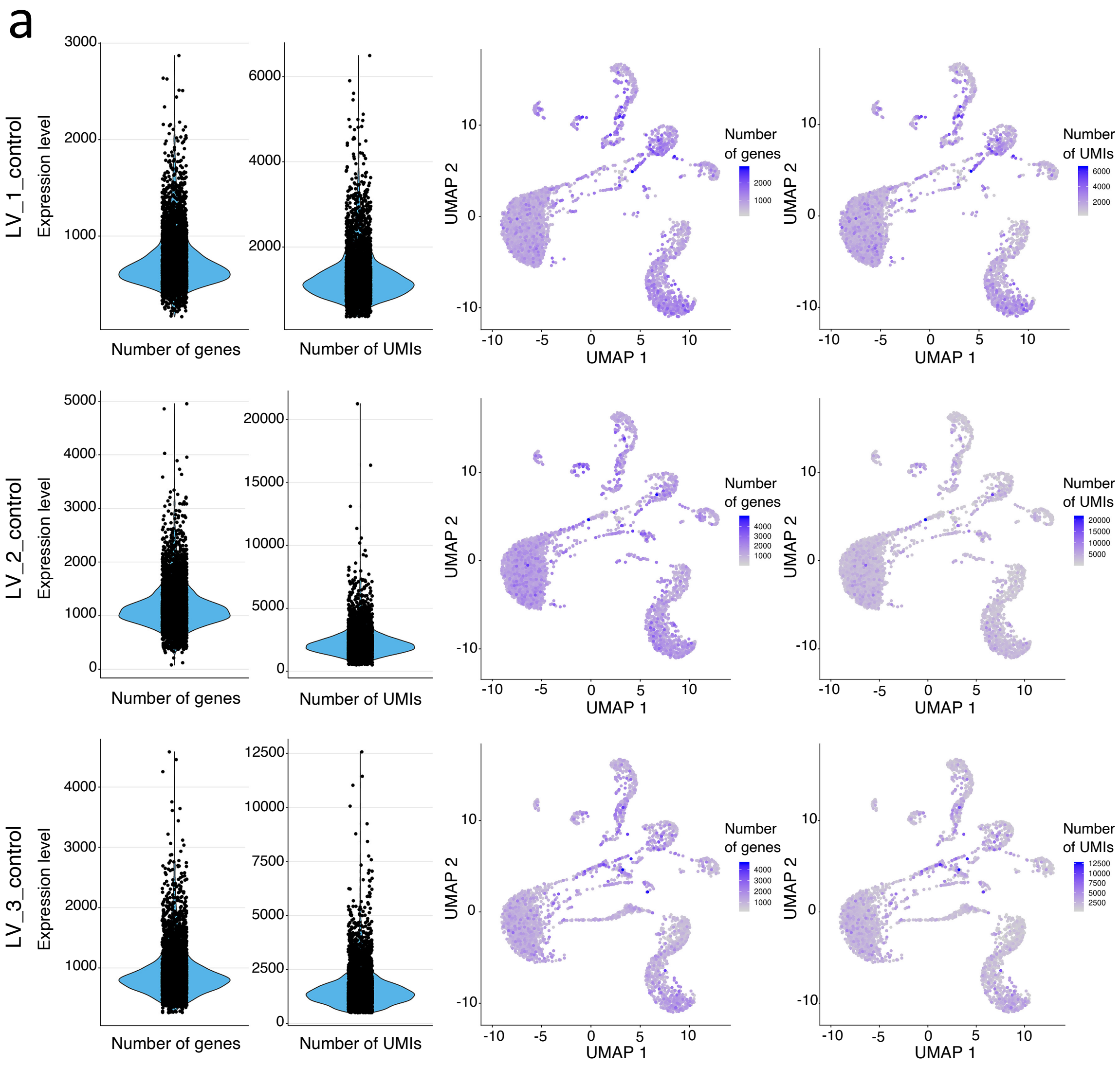
a
LV_1_control
LV_2_control
LV_3_control
Expression level
Number of genes
Number of UMIs
UMAP 1
UMAP 2
Number of genes
Number of UMIs

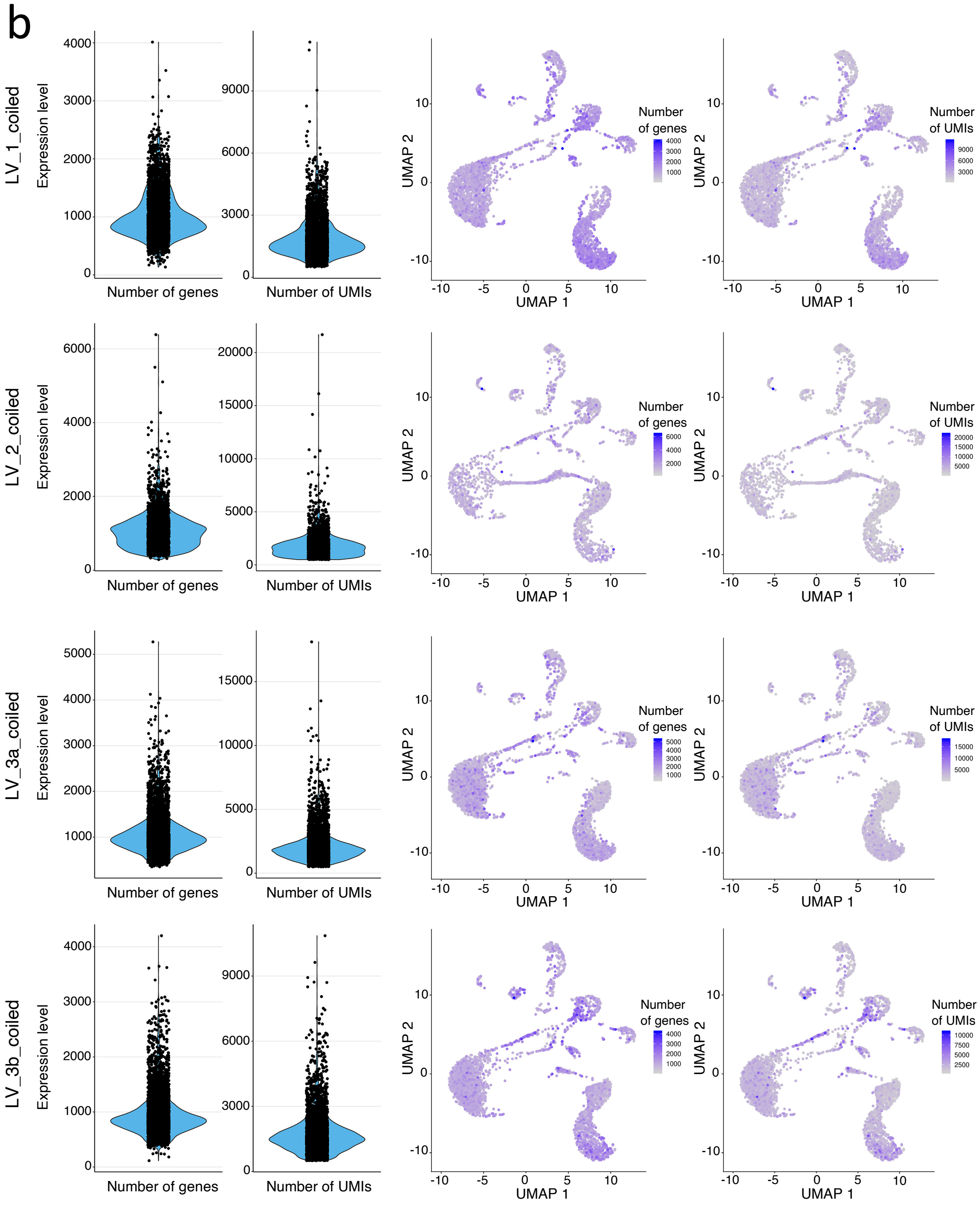

Figure S10. Number of genes or unique molecular identifiers (UMIs) for control (a) and coiled (b) samples. In the violin plots (left panel), each nucleus is represented by a dot. The blue violin represents the distribution. In the Uniform Manifold Approximation and Projection (UMAP) plots (right panel), each nucleus is represented by a dot. The colors correspond to the number of genes or UMIs measured per nucleus.

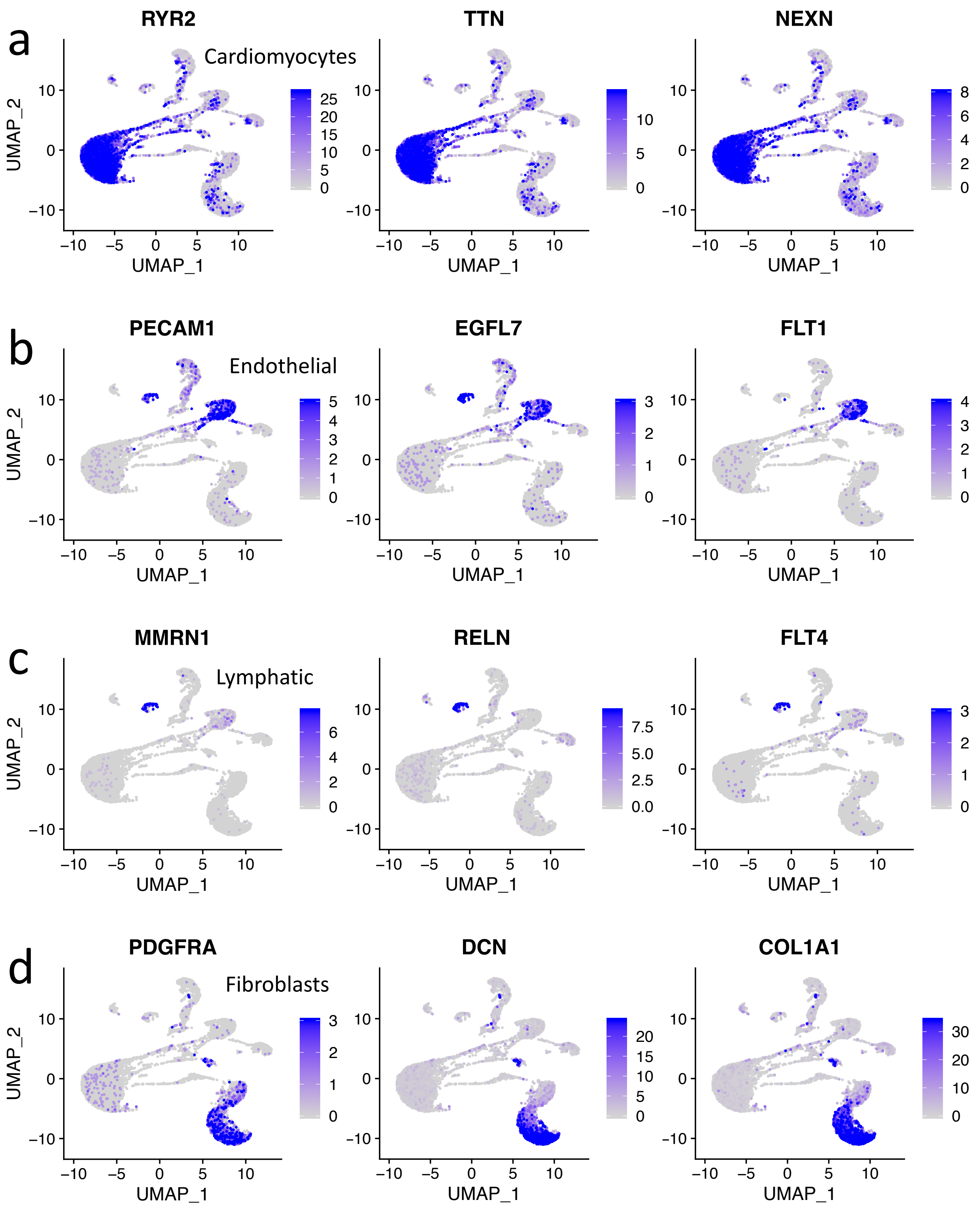
a
RYR2
TTN
NEXN
Cardiomyocytes
b
PECAM1
EGFL7
FLT1
Endothelial
c
MMRN1
RELN
FLT4
Lymphatic
d
PDGFRA
DCN
COL1A1
Fibroblasts
UMAP_1
UMAP_2

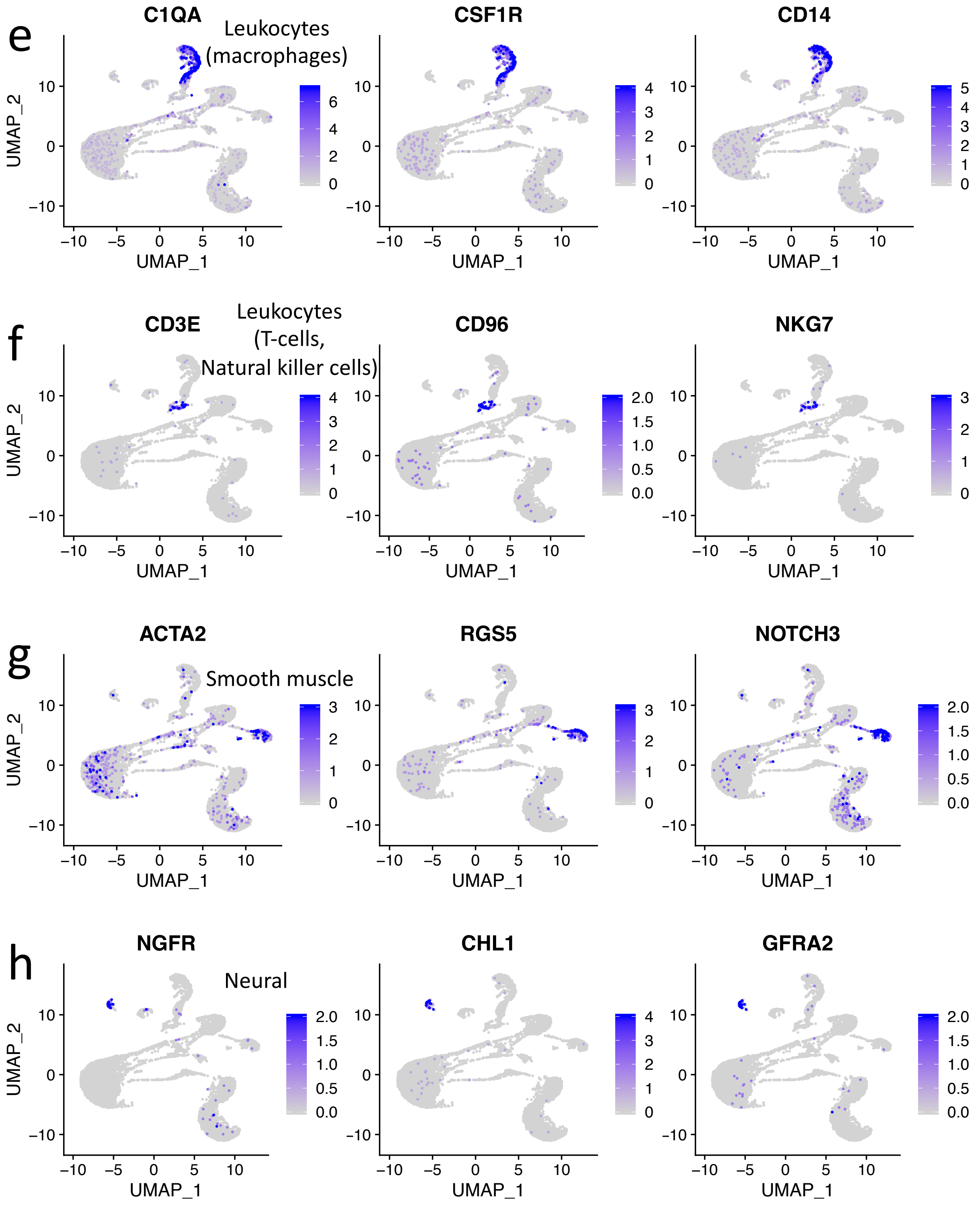

e
C1QA
Leukocytes (macrophages)
CSF1R
CD14
f
CD3E
Leukocytes (T-cells, Natural killer cells)
CD96
NKG7
g
ACTA2
Smooth muscle
RGS5
NOTCH3
h
NGFR
Neural
CHL1
GFRA2
UMAP_1
UMAP_2

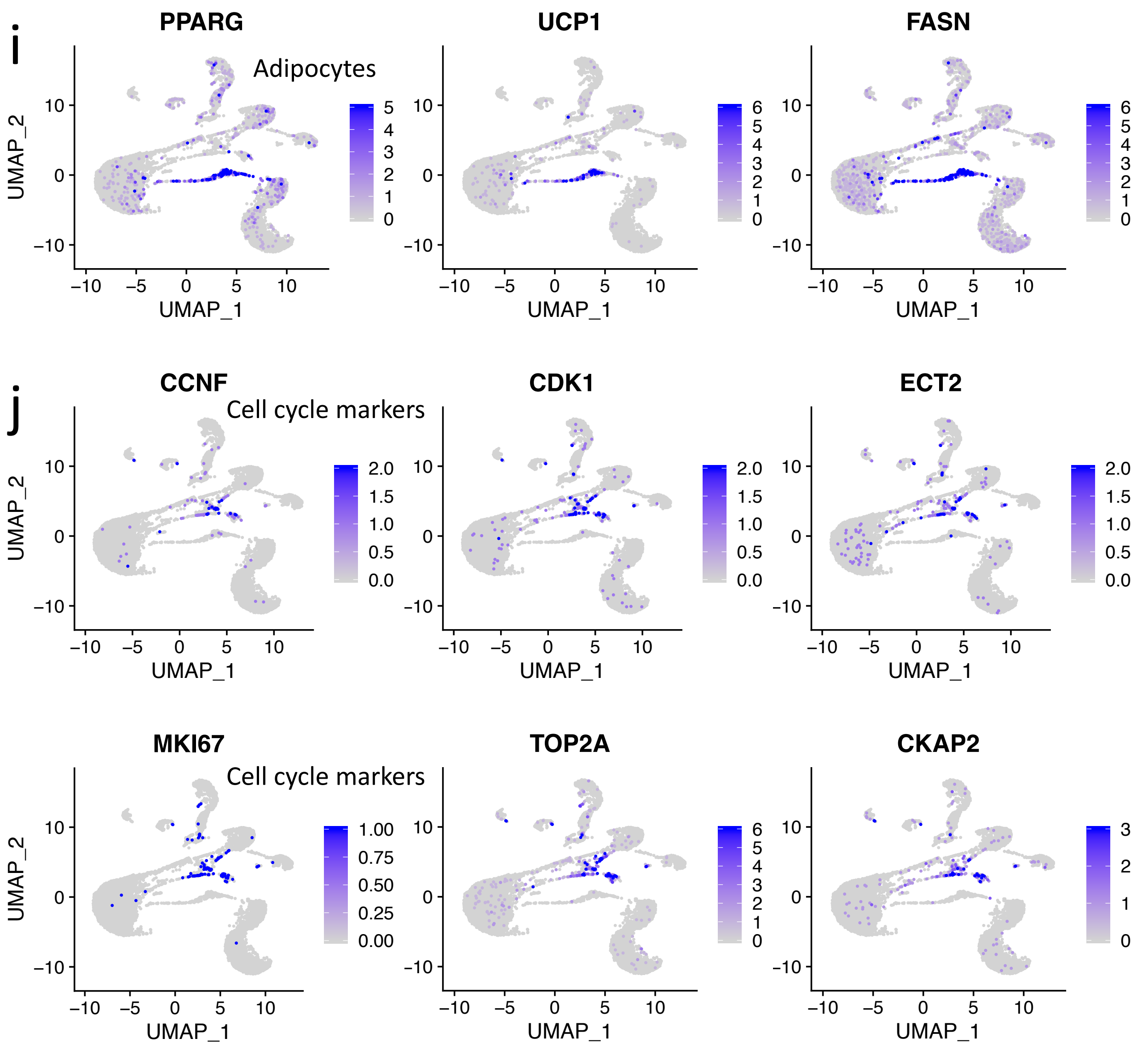

Figure S11. Uniform Manifold Approximation and Projection (UMAP) plots of selected marker genes.

Panel a. *Cardiomyocyte markers:* RYR2 (ryanodine receptor 2; required for cardiac muscle excitation-contraction coupling), TTN (titin; key component in the assembly and functioning of striated muscles), NEXN (nexilin; essential role in the sarcomere integrity of cardiac muscle).

Panel b. *Endothelial cell markers:* PECAM1 (platelet endothelial cell adhesion molecule; expressed in endothelial cells and leukocytes), EGFL7 (epidermal growth factor-like protein 7; expressed in endothelial cells and progenitors during development), FLT1 (vascular endothelial growth factor receptor 1; important role in angiogenesis and vasculogenesis).

Panel c. *Lymphatic endothelial cell markers:* MMRN1 (multimerin 1; lymphatic endothelial marker), RELN (reelin; characteristic for the lymphatic lineage), FLT4 (vascular endothelial growth factor receptor 3; involved in lymphangiogenesis and maintenance of the lymphatic endothelium).

Panel d. *Fibroblast markers:* PDFGRA (platelet-derived growth factor receptor A; required in developing cardiac fibroblasts), DCN (decorin; extracellular matrix protein, considered a pan-fibroblast marker), COL1A1 (collagen type 1, alpha 1; component of connective tissues, secreted by fibroblasts).

Panel e. *Leukocyte markers (including macrophages):* C1QA (complement C1q subcomponent subunit A; major constituent of the human complement system), CSF1R (macrophage colony-stimulating factor 1 receptor; essential roles in mononuclear phagocytes, such as macrophages and monocytes), CD14 (cluster of differentiation 14; expressed mostly by macrophages as part of the innate immune system)

Panel f. *Leukocyte markers (including T-cells, natural killer cells):* CD3E (T-cell surface glycoprotein CD3 epsilon chain; essential role in correct T-cell development), CD96 (cluster of differentiation 96; immunoglobulin superfamily, expressed on T cells and NK cells), NKG7 (Natural killer cell granule protein 7; integral membrane protein on cytotoxic granules of quiescent NK and CD8 T cells).

Panel g. *Smooth muscle cell makers:* ACTA2 (actin alpha 2, smooth muscle; smooth muscle actin), RGS5 (regulator of G protein signaling 5; abundantly expressed in vascular smooth muscle cells), NOTCH3 (Notch receptor 3; key role in the function and survival of vascular smooth muscle cells).

Panel h. *Neural cell markers:* NGFR (nerve growth factor receptor; receptor subunit for multiple neurotrophins), CHL1 (cell adhesion molecule L1 like; expressed in the peripheral and central nervous systems, plays a role in nervous system development and synaptic plasticity), GFRA2 (GDNF family receptor alpha-2; glial cell line-derived neurotrophic factor with key roles in neuron survival and differentiation).

Panel i. *Adipocyte markers:* PPARG (peroxisome proliferator activated receptor gamma; transcriptional regulator of adipocyte differentiation), UCP1 (thermogenin; mitochondrial protein responsible for thermogenesis by uncoupling oxidative phosphorylation), FASN (fatty acid synthase; multi-enzyme protein that catalyzes fatty acid synthesis).

Panel j. *Cell cycle marker genes:* CCNF (G2/mitotic-specific cyclin-F; regulator of cell cycle transitions), CDK1 (cyclin-dependent kinase 1; serine/threonine kinase and key player in cell cycle regulation), ECT2 (epithelial cell transforming 2; important role in the regulation of cytokinesis), MKI67 (marker of proliferation Ki-67; commonly used to detect and quantify proliferating cells), TOP2A (DNA topoisomerase 2-alpha; maximally expressed in the G_2 and M phases of the cell cycle), CKAP2 (cytoskeleton-associated protein 2; involved in mitotic progression).

Table S3. Nucleic numbers, per cluster and sample/condition. Cell type assignments as per Gene Set Variation Analysis (GSVA) and the expression of characteristic marker genes.

Sample	Cluster	Nuclei number per sample	Nuclei number per condition
LV_1_control	c0 cardiomyocytes	1728	6044
LV_2_control		2357	
LV_3_control		1959	
LV_1_coiled		1206	5705
LV_2_coiled		337	
LV_3_coiled		2375	
LV_4_coiled		1787	
LV_1_control	c1 fibroblasts	464	1342
LV_2_control		494	
LV_3_control		384	
LV_1_coiled		789	2348
LV_2_coiled		257	
LV_3_coiled		799	
LV_4_coiled		503	
LV_1_control	c2 fibroblasts	302	1043
LV_2_control		317	
LV_3_control		424	
LV_1_coiled		404	2472
LV_2_coiled		618	
LV_3_coiled		665	
LV_4_coiled		785	
LV_1_control	c3 cardiomyocytes	584	1266
LV_2_control		484	
LV_3_control		198	
LV_1_coiled		770	1335
LV_2_coiled		93	
LV_3_coiled		302	
LV_4_coiled		170	
LV_1_control	c4 leukocytes	225	814
LV_2_control		239	
LV_3_control		350	
LV_1_coiled		337	1042
LV_2_coiled		189	
LV_3_coiled		269	
LV_4_coiled		247	
LV_1_control	c5 endothelial cells	239	639
LV_2_control		194	
LV_3_control		206	
LV_1_coiled		308	1183
LV_2_coiled		309	
LV_3_coiled		196	
LV_4_coiled		370	
LV_1_control	c6 smooth muscle cells	168	425
LV_2_control		122	

LV_3_control		135	
LV_1_coiled		169	632
LV_2_coiled		105	
LV_3_coiled		247	
LV_4_coiled		111	
LV_1_control	c7* undefined (mitosis-related)	69	360
LV_2_control		96	
LV_3_control		195	
LV_1_coiled		109	405
LV_2_coiled		90	
LV_3_coiled		88	
LV_4_coiled		118	
LV_1_control	c8 adipocytes	5	149
LV_2_control		27	
LV_3_control		117	
LV_1_coiled		0	467
LV_2_coiled		332	
LV_3_coiled		19	
LV_4_coiled		116	
LV_1_control	c9 adipocytes	1	91
LV_2_control		0	
LV_3_control		90	
LV_1_coiled		5	491
LV_2_coiled		484	
LV_3_coiled		1	
LV_4_coiled		1	
LV_1_control	c10 cardiomyocytes	47	228
LV_2_control		72	
LV_3_control		109	
LV_1_coiled		60	316
LV_2_coiled		98	
LV_3_coiled		89	
LV_4_coiled		69	
LV_1_control	c11 leukocytes	90	201
LV_2_control		78	
LV_3_control		33	
LV_1_coiled		99	215
LV_2_coiled		23	
LV_3_coiled		61	
LV_4_coiled		32	
LV_1_control	c12 lymphatic endothelial cells	24	163
LV_2_control		53	
LV_3_control		86	
LV_1_coiled		16	234
LV_2_coiled		61	
LV_3_coiled		47	
LV_4_coiled		110	
LV_1_control	c13	77	138

LV_2_control	neural cells	37	
LV_3_control		24	
LV_1_coiled		81	187
LV_2_coiled		48	
LV_3_coiled		27	
LV_4_coiled		31	
LV_1_control	c14 undefined	5	52
LV_2_control		11	
LV_3_control		36	
LV_1_coiled		28	157
LV_2_coiled		54	
LV_3_coiled		54	
LV_4_coiled		21	
LV_1_control	c15 endothelial cells (mitosis-related)	12	37
LV_2_control		13	
LV_3_control		12	
LV_1_coiled		14	56
LV_2_coiled		16	
LV_3_coiled		13	
LV_4_coiled		13	
LV_1_control	total	4040	12992
LV_2_control		4594	
LV_3_control		4358	
LV_1_coiled		4395	17245
LV_2_coiled		3114	
LV_3_coiled		5252	
LV_4_coiled		4484	

* c7 subclusters in Figure S12, Table S4.

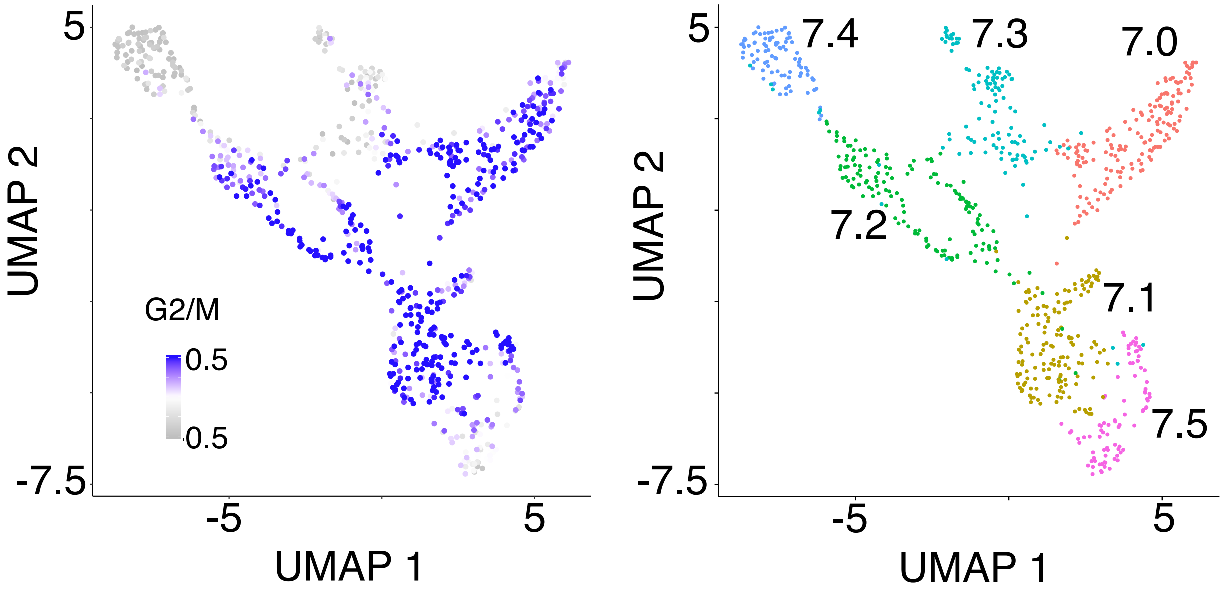

Figure S12. UMAP cluster 7, re-clustered and displayed with new coordinates: Expression of cell cycle-related genes (G2 phase and mitosis; left panel) and colour-coded subclusters (right panel). Tentative cell type assignments as per Gene Set Variation Analysis (GSVA): adipocytes (7.4), cardiomyocytes (7.0, 7.3), fibroblasts (7.1, 7.5), undefined (7.2).

Table S4. UMAP cluster 7, re-clustered: Nucleic numbers, per cluster and sample/condition. Tentative cell type assignments as per Gene Set Variation Analysis (GSVA)

<table>
<tr><th>Sample</th><th>Cluster</th><th>Nuclei number per sample</th><th>Nuclei number per condition</th></tr>
<tr><td>LV_1_control</td><td rowspan="7">C7.0
cardiomyocytes</td><td>24</td><td rowspan="3">88</td></tr>
<tr><td>LV_2_control</td><td>30</td></tr>
<tr><td>LV_3_control</td><td>34</td></tr>
<tr><td>LV_1_coiled</td><td>12</td><td rowspan="4">77</td></tr>
<tr><td>LV_2_coiled</td><td>8</td></tr>
<tr><td>LV_3_coiled</td><td>28</td></tr>
<tr><td>LV_4_coiled</td><td>29</td></tr>
<tr><td>LV_1_control</td><td rowspan="7">C7.1
fibroblasts</td><td>10</td><td rowspan="3">50</td></tr>
<tr><td>LV_2_control</td><td>21</td></tr>
<tr><td>LV_3_control</td><td>19</td></tr>
<tr><td>LV_1_coiled</td><td>17</td><td rowspan="4">105</td></tr>
<tr><td>LV_2_coiled</td><td>15</td></tr>
<tr><td>LV_3_coiled</td><td>31</td></tr>
<tr><td>LV_4_coiled</td><td>42</td></tr>
<tr><td>LV_1_control</td><td rowspan="7">C7.2
undefined</td><td>3</td><td rowspan="3">89</td></tr>
<tr><td>LV_2_control</td><td>2</td></tr>
<tr><td>LV_3_control</td><td>84</td></tr>
<tr><td>LV_1_coiled</td><td>7</td><td rowspan="4">63</td></tr>
<tr><td>LV_2_coiled</td><td>51</td></tr>
<tr><td>LV_3_coiled</td><td>2</td></tr>
<tr><td>LV_4_coiled</td><td>3</td></tr>
<tr><td>LV_1_control</td><td rowspan="7">C7.3
cardiomyocytes</td><td>15</td><td rowspan="3">57</td></tr>
<tr><td>LV_2_control</td><td>19</td></tr>
<tr><td>LV_3_control</td><td>23</td></tr>
<tr><td>LV_1_coiled</td><td>23</td><td rowspan="4">60</td></tr>
<tr><td>LV_2_coiled</td><td>4</td></tr>
<tr><td>LV_3_coiled</td><td>10</td></tr>
<tr><td>LV_4_coiled</td><td>23</td></tr>
<tr><td>LV_1_control</td><td rowspan="7">C7.4
adipocytes</td><td>6</td><td rowspan="3">41</td></tr>
<tr><td>LV_2_control</td><td>8</td></tr>
<tr><td>LV_3_control</td><td>27</td></tr>
<tr><td>LV_1_coiled</td><td>28</td><td rowspan="4">49</td></tr>
<tr><td>LV_2_coiled</td><td>9</td></tr>
<tr><td>LV_3_coiled</td><td>5</td></tr>
<tr><td>LV_4_coiled</td><td>7</td></tr>
<tr><td>LV_1_control</td><td rowspan="7">C7.5
fibroblasts</td><td>11</td><td rowspan="3">35</td></tr>
<tr><td>LV_2_control</td><td>16</td></tr>
<tr><td>LV_3_control</td><td>8</td></tr>
<tr><td>LV_1_coiled</td><td>22</td><td rowspan="4">51</td></tr>
<tr><td>LV_2_coiled</td><td>3</td></tr>
<tr><td>LV_3_coiled</td><td>12</td></tr>
<tr><td>LV_4_coiled</td><td>14</td></tr>
</table>

Table S5. Nucleic counts per cell type, in control and coiled fetuses. Per sample (n = 7 samples). Include tentatively assigned cell types from cluster c7. Sample LV_2_coiled was considered an outlier and not included in Figure 4.

Counts	Adipo	Cardio	Endo	Fibro	Leuko	Lymph	Neural	Smooth	Undef	Total
LV_1_control	12	2398	251	787	315	24	77	168	8	4040
LV_2_control	35	2962	207	848	317	53	37	122	13	4594
LV_3_control	234	2323	218	835	383	86	24	135	120	4358
LV_1_coiled	33	2071	322	1232	436	16	81	169	35	4395
LV_2_coiled	825	540	325	893	212	61	48	105	105	3114
LV_3_coiled	25	2804	209	1507	330	47	27	247	56	5252
LV_4_coiled	124	2078	383	1344	279	110	31	111	24	4484

Adipo, adipocytes; cardio, cardiomyocytes; endo, endothelial cells; fibro, fibroblasts; leuko, leukocytes; lymph, lymphatic endothelial cells; neural, neural cells; smooth, smooth muscle cells; undef, undefined; LV, left ventricle.

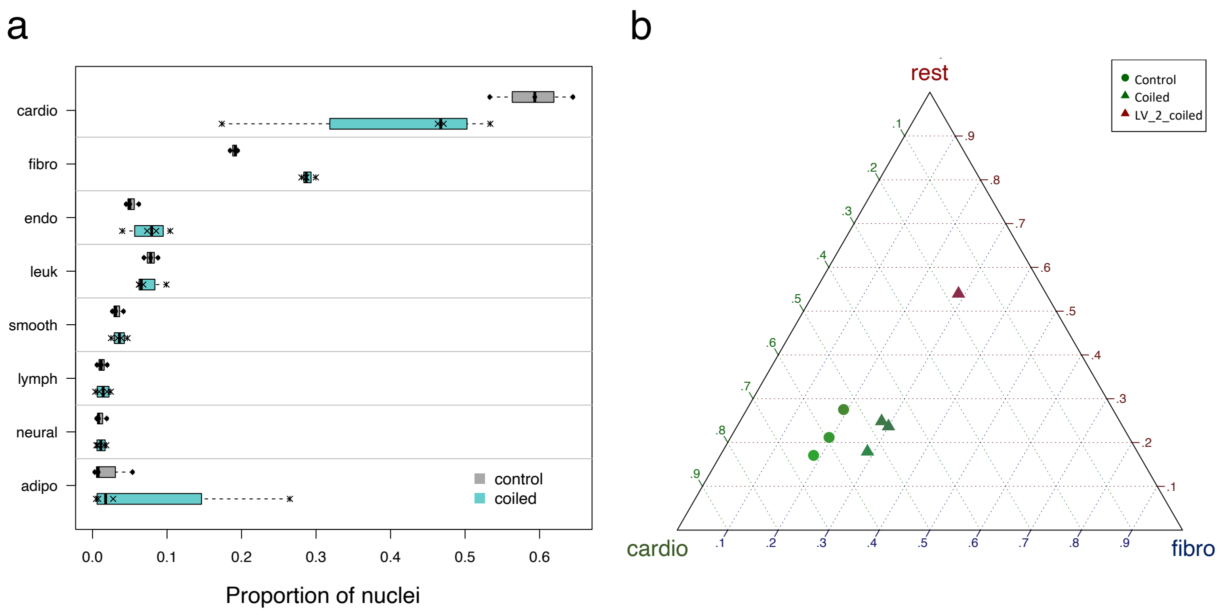

Figure S13. (a) Boxplots (proportions of nuclei) in coiled (hypoplastic) and control left ventricles. Sample LV_2_coiled, containing a disproportionately large fraction of adipocytes, was included in the analysis. (b) Proportions of cardiomyocyte nuclei, fibroblast nuclei, and "rest" (adipocytes, endothelial cells, lymphatic endothelial cells, leukocytes, neural cells, smooth muscle cells; Table S3). In a ternary plot of proportions, each point has three coordinates which sum up to one. Lines of constant values (isolines) from the "cardiomyocytes" axis run diagonally to the "fibroblast" axis, whose isolines run diagonally to the "rest" axis. The isolines of the "rest" axis are horizontal lines to the "myocyte" axis. Sample LV_2_coiled is a clear outlier: it has a similar "fibroblast" proportion to the other three coiled samples, but has a much lower proportion of cardiomyocyte nuclei and much higher proportion of "rest" nuclei. This high value for "rest" nuclei was mainly due to adipocytes. Numerical data in Table S5.

Table S6. Fisher's exact test, differential abundance of nuclei in pooled control (n = 3) versus pooled coiled (n = 3) samples.

	Odds ratio	p-value	Nuclei coiled	Nuclei controls	FoldChange	Absolute FoldChange	Bias	Cell type	Bonferroni corrected p-value	Significance
Cardio	0.669372	1.87E-60	6953	7683	-1.49394	1.493938	cn	Cardiomyocytes (14636 cells)	1.68E-59	coil biased
Undef	0.747822	0.023491	115	141	-1.33722	1.337216	cn	Undefined (256 cells)	0.211423	not significant
Fibro	1.730952	3.92E-81	4083	2470	1.730952	1.730952	coil	Fibroblasts (6553 cells)	3.53E-80	coil biased
Smooth	1.145471	0.040662	527	425	1.145471	1.145471	coil	Smooth muscle cells (952 cells)	0.365959	not significant
Endo	1.259887	9.55E-06	914	676	1.259887	1.259887	coil	Endothelial cell (1590 cells)	8.59E-05	coil biased
Lymph	0.975497	0.826271	173	163	-1.02512	1.025118	cn	Lymphatic cells (336 cells)	1	not significant
Neural	0.925344	0.545689	139	138	-1.08068	1.080679	cn	Neural cell (277 cells)	1	not significant
Adipo	0.590221	2.7E-08	182	281	-1.69428	1.694282	cn	Adipocytes (463 cells)	2.43E-07	coil biased
Leuko	0.942337	0.198926	1045	1015	-1.06119	1.061191	cn	Leukocytes (2060 cells)	1	not significant

Adipo, adipocytes; cardio, cardiomyocytes; endo, endothelial cells; fibro, fibroblasts; leuko, leukocytes; lymph, lymphatic endothelial cells; neural, neural cells; smooth, smooth muscle cells; undef, undefined; cn, control.

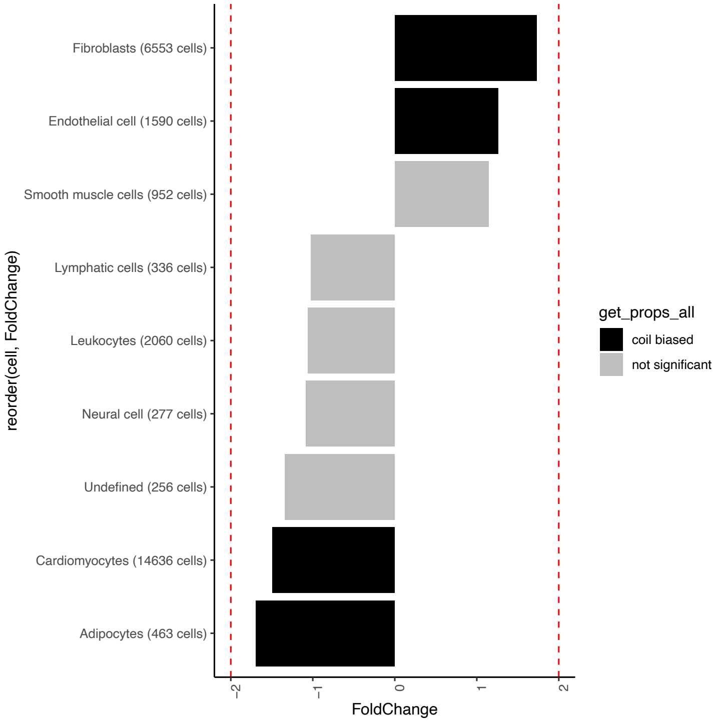

Figure S14. Fisher's exact test, differential abundance of nuclei in pooled control (n = 3) versus pooled coiled (n = 3) samples. The change in endothelial cells, cardiomyocytes and adipocytes was not significant by *scCODA* or *DirichletReg*. Numerical data in Table S6.

Table S7. Nucleic proportions per cell type, in pooled control versus pooled coiled samples (multinomial proportion analyses). n = 6 samples: Sample LV_2_coiled contained a disproportionately large fraction of adipocytes, and was therefore excluded; see above. Undefined nuclei were excluded. (a) Empirical proportions. (b) *ScCODA* predicted proportions.

a

Empirical proportions (%)	Adipo	Cardio	Endo	Fibro	Leuko	Lymph	Neural	Smooth	Total
LV_control	2.178	59.947	5.239	19.143	7.866	1.263	1.070	3.294	100
LV_coiled	1.289	49.975	6.474	28.918	7.401	1.225	0.984	3.733	100

b

ScCODA predicted proportions (%)	Adipo	Cardio	Endo	Fibro	Leuko	Lymph	Neural	Smooth	Total
LV_control	1.287	59.105	5.676	20.092	7.904	1.265	1.144	3.527	100
LV_coiled	1.148	52.733	5.064	28.707	7.052	1.129	1.020	3.146	100

The number of cells of a particular cell type in a sample has an upper limit imposed by the total number of cells that were sequenced. For common cell types this will be of the same order of magnitude as the total number of cells sequenced. One assumption of modelling counts, using Poisson or Negative Binomial (Gamma-Poisson) distributions, is that there is, in principle, no upper limit to the counts that may occur. Hence the counts of different cell types are best represented as a proportion of the total number of cells sequenced in the sample, i.e., as compositional data/proportion of the total, rather than pure counts. Two methods were used to analyze the composition of myocardium, based on the different cell types being modelled by a multinomial distribution that was converted to multinomial proportions by being represented by a Dirichlet-Multinomial distribution. Endothelial cell abundance was similar between controls and coiled samples, but there was wider variation in the other three cell types. In the case of adipocytes this may be a sampling issue, due to true regional variation in adipose tissue distribution in large mammalian heart that can often be seen by direct inspection of the surface of the heart. Mice have only minor amounts of epicardial adipose tissue [12]. Sample LV_2_coiled was atypical with the smallest number of total nuclei, largest number of adipocytes and fewest cardiomyocytes. The limited number of replicates (3 controls, 4 coiled) mean we cannot be certain if sample LV_2_coiled (Figure S13, ternary diagram) represents the effects of the underlying disease process or is just a consequence of a spatial sampling issue resulting in many adipocytes and few cardiomyocytes. We have adopted a conservative approach and treated this sample as a likely outlier representing a region with a deposit of adipose tissue, and excluded it from inferences on changes in cell composition that could be attributed to changes secondary to low left heart flow. Results from *scCODA* show a credible $\log_2$-fold change of 0.515, i.e., a 1.43 fold increase in fibroblasts. This was with a reference cell type of neural cells, but the estimate was similar ($\log_2$-fold change 0.49, in absolute terms 1.41 fold) if smooth muscle was used as the reference. *DirichletReg* using a maximum likelihood procedure, estimated a 1.48 fold increase in fibroblasts (95% confidence intervals 1.1 - 2.0). Hence using different reference cell types and a different estimation technique are reassuringly consistent in predicting a ~1.4 fold increase in fibroblasts.

Abbreviations: Adipo, adipocytes; cardio, cardiomyocytes; endo, endothelial cells; fibro, fibroblasts; leuko, leukocytes; lymph, lymphatic endothelial cells; neural, neural cells; smooth, smooth muscle cells; LV, left ventricle.

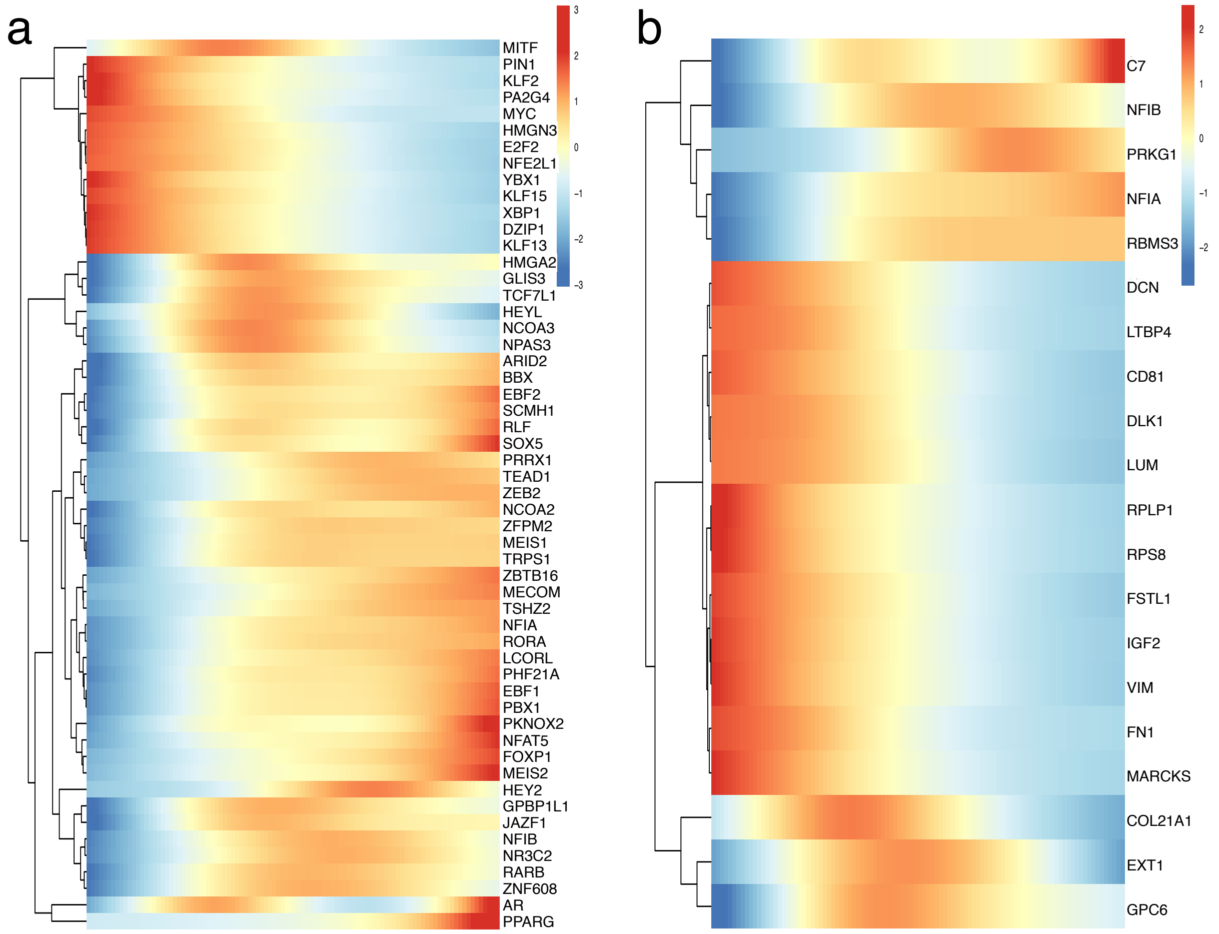

Figure S15. Fibroblast manifold associated gene expression. Heatmaps representing gene trends over pseudotime c1 to c2: (a) Transcription factor expression profiles. (b) Top expressed genes in human fetal heart stromal cells (https://descartes.brotmanbaty.org/bbi/human-gene-expression-during-development/cell/stromal/in/heart).

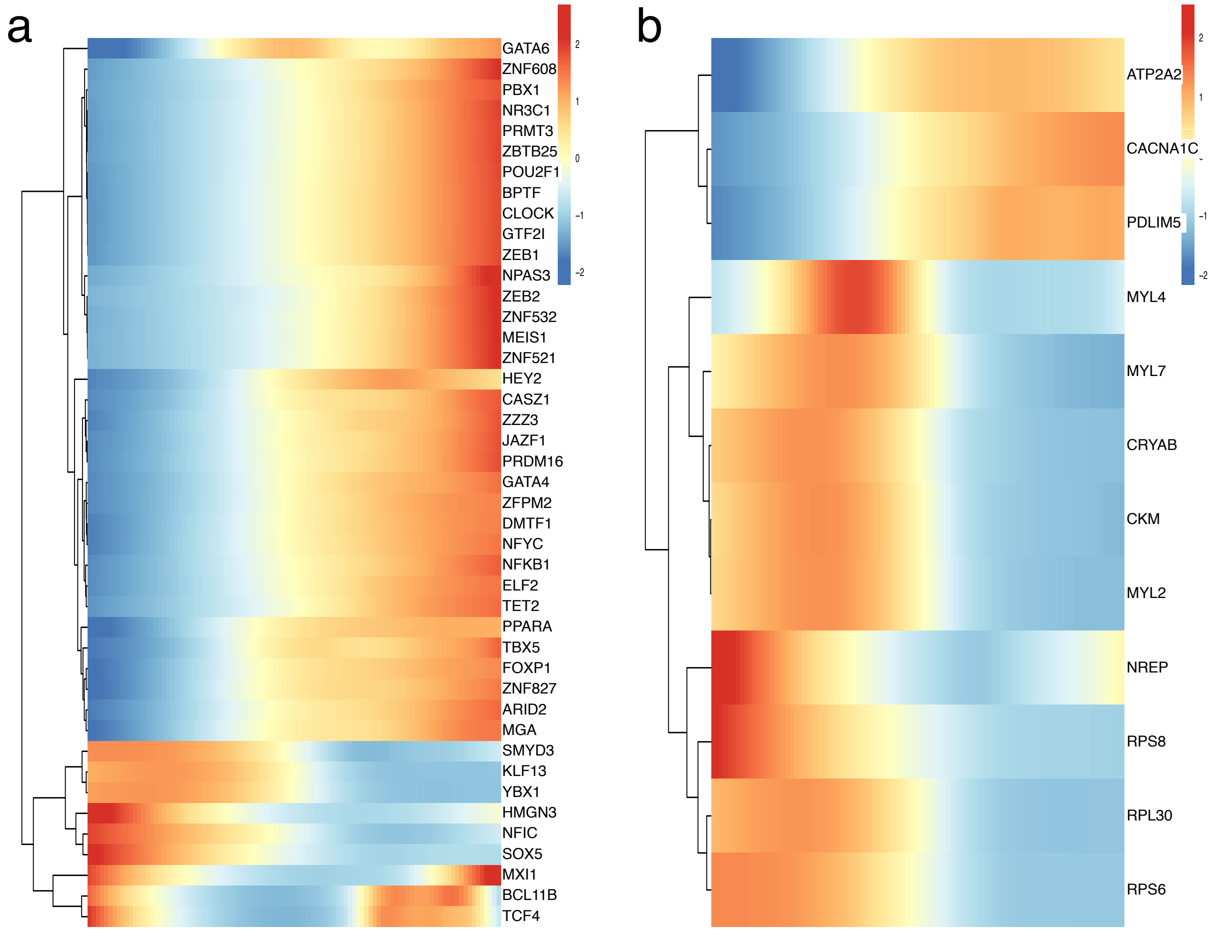

Figure S16. Cardiomyocyte manifold associated gene expression. Heatmaps representing gene trends over pseudotime from c10 to c0: (a) Transcription factor expression profiles. (b) Top expressed genes in human fetal cardiomyocytes (https://descartes.brotmanbaty.org/bbi/human-gene-expression-during-development/cell/cardiomyocytes/in/heart).

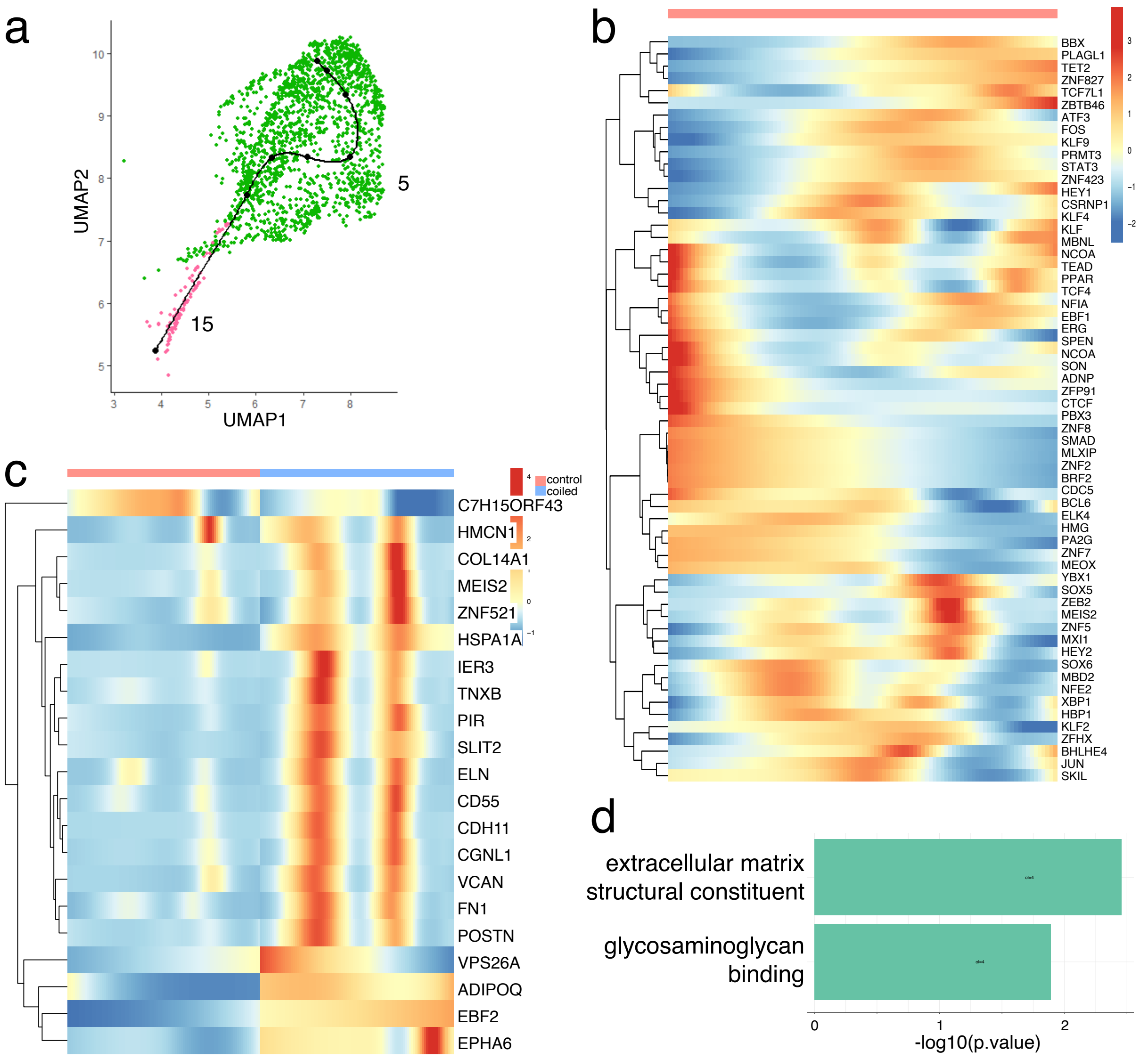

Figure S17. Endothelial manifold (a) associated gene expression. Heatmaps representing gene trends over pseudotime from c15 to c5: (b) Transcription factor expression profiles. (c) Heatmap representing differentially expressed genes over pseudotime, controls (left) and coiled (hypoplastic) left ventricles (right). (d) Gene ontology (GO) enrichment analysis of differentially expressed genes.

a

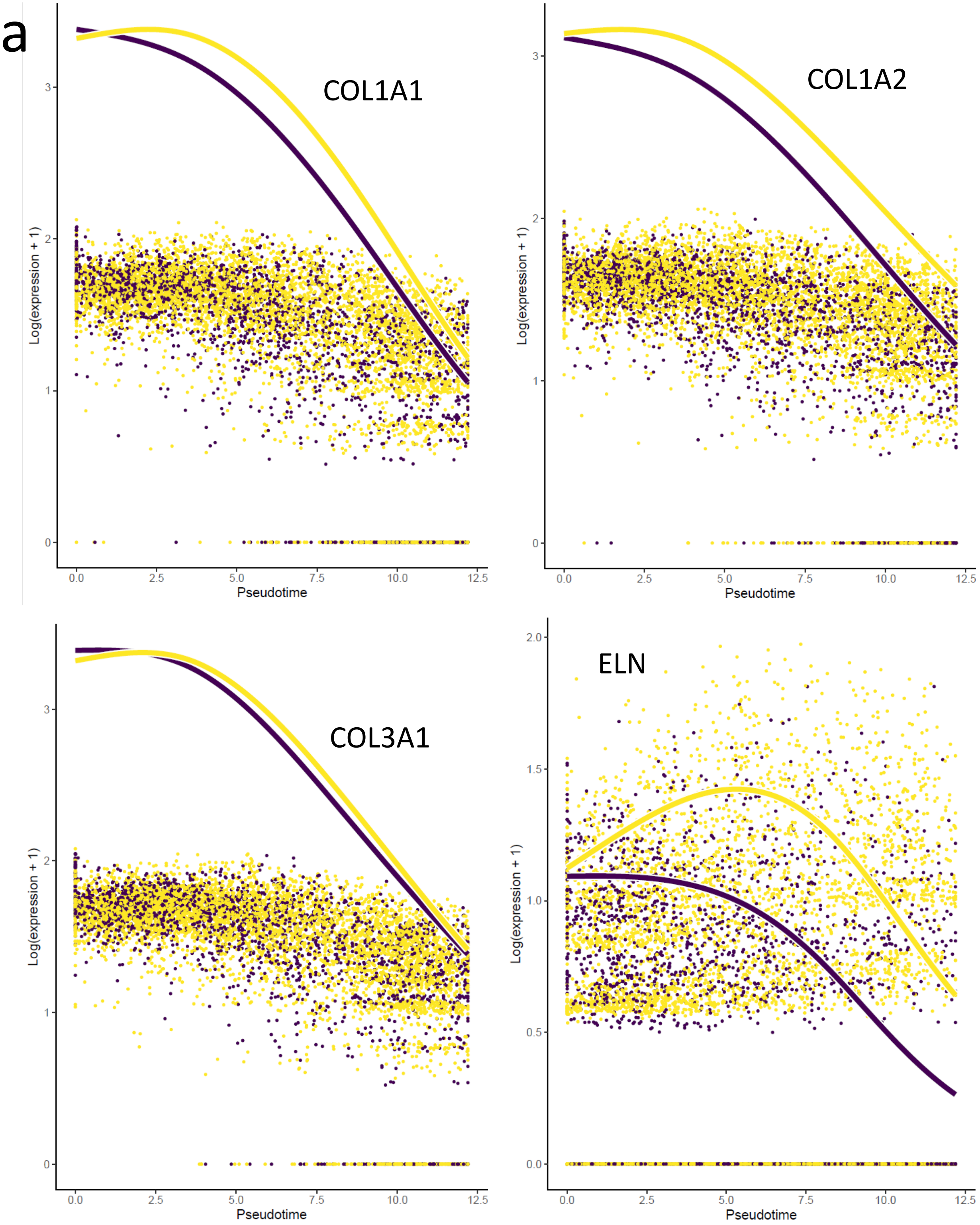
COL1A1
COL1A2
COL3A1
ELN
Log(expression + 1)
Pseudotime
0.0
2.5
5.0
7.5
10.0
12.5
0
1
2
3
0.0
0.5
1.0
1.5
2.0

b

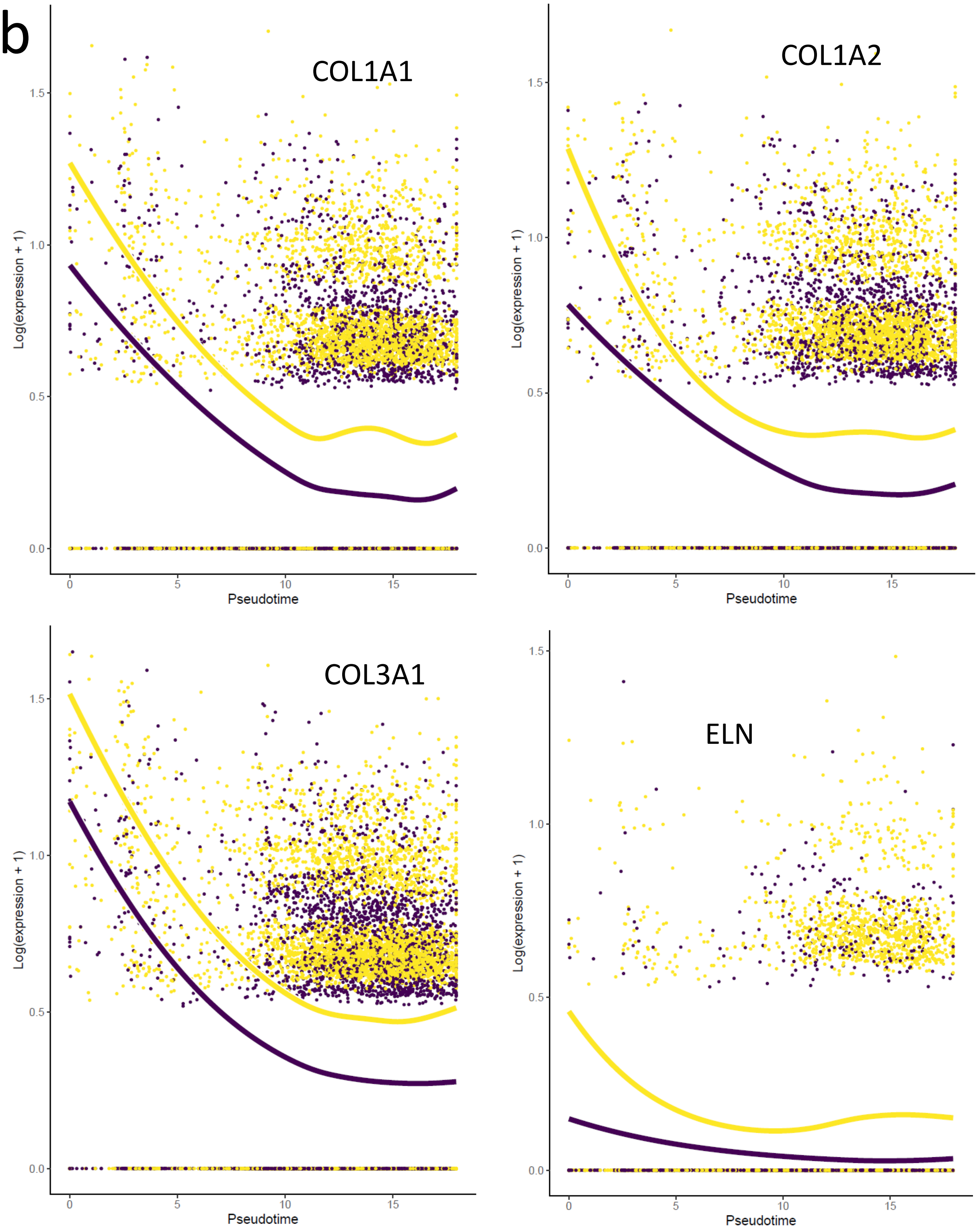

COL1A1
COL1A2
COL3A1
ELN
Log(expression + 1)
Pseudotime
0.0
0.5
1.0
1.5
0
5
10
15

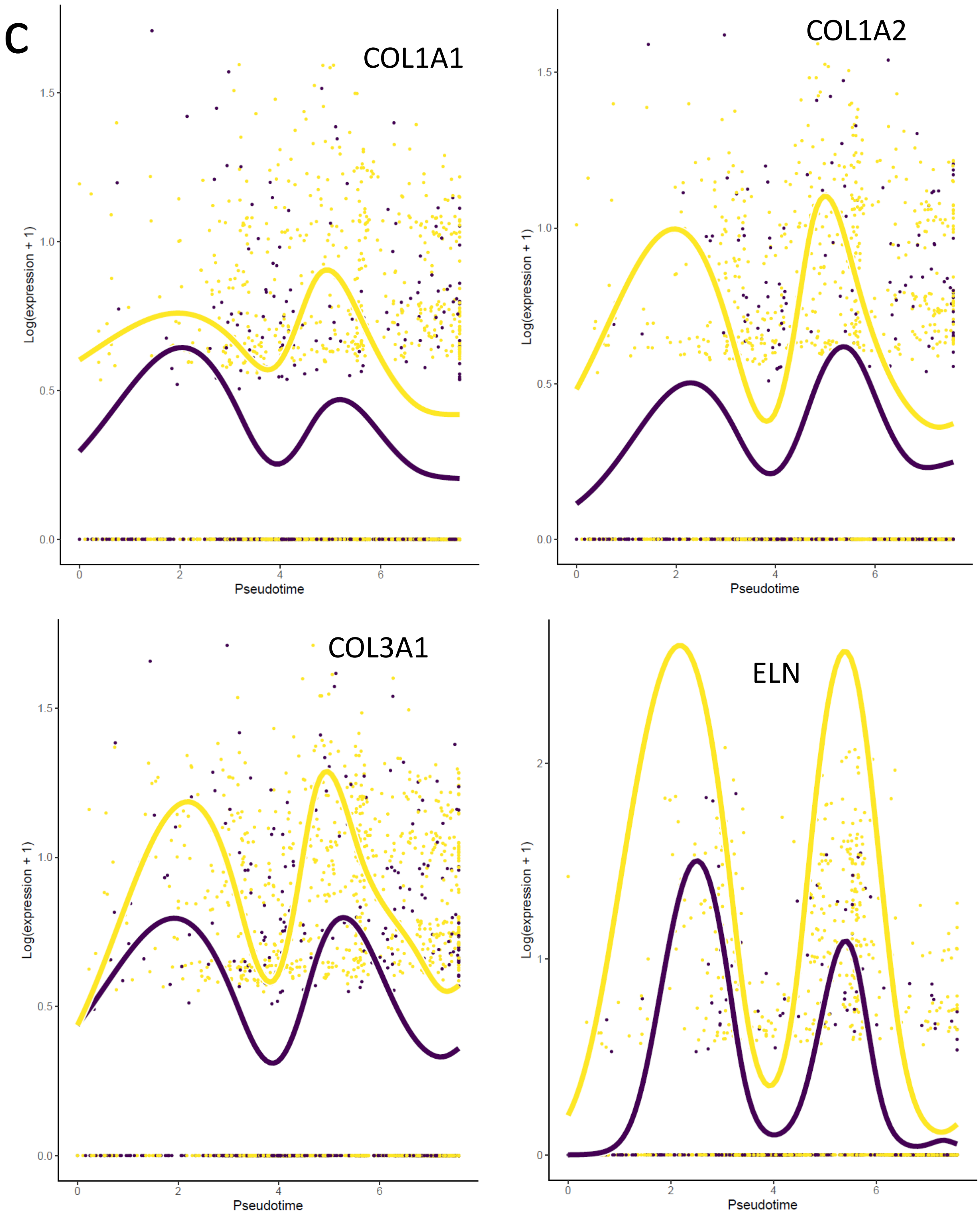

Figure S18. Scatter plots of genes of interest. (a) Fibroblast manifold. (b) Cardiomyocyte manifold. (c) Endothelial manifold. Yellow: coiled samples; black: controls.

Supplementary discussion:

Our CellChat [13] analysis suggested important changes in FGF and VEGF signaling (Figure 7). FGF2 signaling through FGFR1 has been found to be protective against endothelial-to-mesenchymal transition (endMT) in a wide range of endothelial cells [14, 15] and inhibit endMT in the context of fibrosis [16] and atherosclerosis [17]. The molecular basis for this FGF2 antagonism of endMT, even TGF-beta/Smad driven, remains to be determined. In our samples, the target of this FGF2 antagonism of endMT was particularly focused on endothelial cells (Figure 7). The importance of endMT was first demonstrated in embryonic cardiac valve formation [18] and the role of VEGFA in this process was determined later [19]. Subsequently VEGFA's antagonism of TGF-beta [20] and Notch1 [21] endMT signaling was demonstrated in valve progenitors. In our samples, lymphatic endothelial cells had a decrease in VEGFR3 (*FLT4*) and an increase in VEGFR1 (*FLT1*), whereas endothelial cells had an increase in VEGFR2 (*KDR*) in response to the lower fluid shear stress. VEGFA acting through VEGFR2 is anti-endMT [22-24]. The subtle increase in *VEGFA* expression in cardiomyocytes could further enhance anti-endMT signaling in endothelial cells.

Hence, in our hypoplastic myocardial samples, which have features of endMT already having occurred, the CellChat algorithm has detected anti-endMT responses, in the form of changes in FGF and VEGF signaling.

Supplementary references:

1. Pinto AR, Ilinykh A, Ivey MJ, Kuwabara JT, D'Antoni ML, Debuque R, Chandran A, Wang L, Arora K, Rosenthal NA and Tallquist MD. Revisiting Cardiac Cellular Composition. *Circ Res*. 2016;118:400-9.
2. Bergmann O, Zdunek S, Felker A, Salehpour M, Alkass K, Bernard S, Sjostrom SL, Szewczykowska M, Jackowska T, Dos Remedios C, Malm T, Andra M, Jashari R, Nyengaard JR, Possnert G, Jovinge S, Druid H and Frisen J. Dynamics of Cell Generation and Turnover in the Human Heart. *Cell*. 2015;161:1566-75.
3. Banerjee I, Fuseler JW, Price RL, Borg TK and Baudino TA. Determination of cell types and numbers during cardiac development in the neonatal and adult rat and mouse. *Am J Physiol Heart Circ Physiol*. 2007;293:H1883-91.
4. Cui Y, Zheng Y, Liu X, Yan L, Fan X, Yong J, Hu Y, Dong J, Li Q, Wu X, Gao S, Li J, Wen L, Qiao J and Tang F. Single-Cell Transcriptome Analysis Maps the Developmental Track of the Human Heart. *Cell Rep*. 2019;26:1934-1950 e5.
5. DeLaughter DM, Bick AG, Wakimoto H, McKean D, Gorham JM, Kathiriya IS, Hinson JT, Homsy J, Gray J, Pu W, Bruneau BG, Seidman JG and Seidman CE. Single-Cell Resolution of Temporal Gene Expression during Heart Development. *Dev Cell*. 2016;39:480-490.
6. Li G, Xu A, Sim S, Priest JR, Tian X, Khan T, Quertermous T, Zhou B, Tsao PS, Quake SR and Wu SM. Transcriptomic Profiling Maps Anatomically Patterned Subpopulations among Single Embryonic Cardiac Cells. *Dev Cell*. 2016;39:491-507.
7. Xiao Y, Hill MC, Zhang M, Martin TJ, Morikawa Y, Wang S, Moise AR, Wythe JD and Martin JF. Hippo Signaling Plays an Essential Role in Cell State Transitions during Cardiac Fibroblast Development. *Dev Cell*. 2018;45:153-169 e6.
8. Hu P, Liu J, Zhao J, Wilkins BJ, Lupino K, Wu H and Pei L. Single-nucleus transcriptomic survey of cell diversity and functional maturation in postnatal mammalian hearts. *Genes Dev*. 2018;32:1344-1357.
9. Litvinukova M, Talavera-Lopez C, Maatz H, Reichart D, Worth CL, Lindberg EL, Kanda M, Polanski K, Heinig M, Lee M, Nadelmann ER, Roberts K, Tuck L, Fasouli ES, DeLaughter DM, McDonough B, Wakimoto H, Gorham JM, Samari S, Mahbubani KT, Saeb-Parsy K, Patone G, Boyle JJ, Zhang H, Zhang H, Viveiros A, Oudit GY, Bayraktar OA, Seidman JG, Seidman CE, Noseda M, Hubner N and Teichmann SA. Cells of the adult human heart. *Nature*. 2020;588:466-472.
10. Tucker NR, Chaffin M, Fleming SJ, Hall AW, Parsons VA, Bedi KC, Jr., Akkad AD, Herndon CN, Arduini A, Papangeli I, Roselli C, Aguet F, Choi SH, Ardlie KG, Babadi M, Margulies KB, Stegmann CM and Ellinor PT. Transcriptional and Cellular Diversity of the Human Heart. *Circulation*. 2020;142:466-482.
11. Wolfien M, Galow AM, Muller P, Bartsch M, Brunner RM, Goldammer T, Wolkenhauer O, Hoeflich A and David R. Single-Nucleus Sequencing of an Entire Mammalian Heart: Cell Type Composition and Velocity. *Cells*. 2020;9.
12. Yamaguchi Y, Cavallero S, Patterson M, Shen H, Xu J, Kumar SR and Sucov HM. Adipogenesis and epicardial adipose tissue: a novel fate of the epicardium induced by mesenchymal transformation and PPARgamma activation. *Proc Natl Acad Sci U S A*. 2015;112:2070-5.
13. Jin S, Guerrero-Juarez CF, Zhang L, Chang I, Ramos R, Kuan CH, Myung P, Plikus MV and Nie Q. Inference and analysis of cell-cell communication using CellChat. *Nat Commun*. 2021;12:1088.
14. Piera-Velazquez S and Jimenez SA. Endothelial to Mesenchymal Transition: Role in Physiology and in the Pathogenesis of Human Diseases. *Physiol Rev*. 2019;99:1281-1324.
15. Xu Y and Kovacic JC. Endothelial to Mesenchymal Transition in Health and Disease. *Annu Rev Physiol*. 2023;85:245-267.
16. Li J, Liu H, Srivastava SP, Hu Q, Gao R, Li S, Kitada M, Wu G, Koya D and Kanasaki K. Endothelial FGFR1 (Fibroblast Growth Factor Receptor 1) Deficiency Contributes Differential Fibrogenic Effects in Kidney and Heart of Diabetic Mice. *Hypertension*. 2020;76:1935-1944.

17. Chen PY, Qin L, Baeyens N, Li G, Afolabi T, Budatha M, Tellides G, Schwartz MA and Simons M. Endothelial-to-mesenchymal transition drives atherosclerosis progression. *J Clin Invest*. 2015;125:4514-28.
18. Markwald RR, Fitzharris TP and Manasek FJ. Structural development of endocardial cushions. *Am J Anat*. 1977;148:85-119.
19. Chang CP, Neilson JR, Bayle JH, Gestwicki JE, Kuo A, Stankunas K, Graef IA and Crabtree GR. A field of myocardial-endocardial NFAT signaling underlies heart valve morphogenesis. *Cell*. 2004;118:649-63.
20. Paruchuri S, Yang JH, Aikawa E, Melero-Martin JM, Khan ZA, Loukogeorgakis S, Schoen FJ and Bischoff J. Human pulmonary valve progenitor cells exhibit endothelial/mesenchymal plasticity in response to vascular endothelial growth factor-A and transforming growth factor-beta2. *Circ Res*. 2006;99:861-9.
21. Yang JH, Wylie-Sears J and Bischoff J. Opposing actions of Notch1 and VEGF in post-natal cardiac valve endothelial cells. *Biochem Biophys Res Commun*. 2008;374:512-6.
22. Shi S, Srivastava SP, Kanasaki M, He J, Kitada M, Nagai T, Nitta K, Takagi S, Kanasaki K and Koya D. Interactions of DPP-4 and integrin beta1 influences endothelial-to-mesenchymal transition. *Kidney Int*. 2015;88:479-89.
23. Shi S, Kanasaki K and Koya D. Linagliptin but not Sitagliptin inhibited transforming growth factor-beta2-induced endothelial DPP-4 activity and the endothelial-mesenchymal transition. *Biochem Biophys Res Commun*. 2016;471:184-90.
24. Illigens BM, Casar Berazaluce A, Poutias D, Gasser R, Del Nido PJ and Friehs I. Vascular Endothelial Growth Factor Prevents Endothelial-to-Mesenchymal Transition in Hypertrophy. *Ann Thorac Surg*. 2017;104:932-939.
