# Supplementary material for: Decreased left heart flow in fetal lambs causes left heart hypoplasia and pro-fibrotic tissue remodeling
Source: Commun Biol. 2023 Jul 22;6:770. doi: 10.1038/s42003-023-05132-2 (PMC10363152; doi:10.1038/s42003-023-05132-2)
Supplement: Supplementary file 2 — Description of Additional Supplementary Files [file 42003_2023_5132_MOESM2_ESM.pdf]

Description of Additional Supplementary Files

File name: Supplementary Data 1
Description: Differential gene expression in bulk RNA-seq data.

File name: Supplementary Data 2
Description: Differentially expressed and associated genes for fibroblast, cardiomyocyte, and endothelial cell manifolds.

File name: Supplementary Data 3
Description: Cluster-specific differentially expressed genes between coiled and control samples.

File name: Supplementary Data 4
Description: Cell Ranger quality control.

File name: Supplementary Data 5
Description: Gene coordinates.

File name: Supplementary Data 6
Description: Cell Metadata.

File name: Supplementary Data 7
Description: Gene set signatures.

File name: Supplementary Data 8
Description: Gene set variation analysis.
