# Supplementary material for: Decreased left heart flow in fetal lambs causes left heart hypoplasia and pro-fibrotic tissue remodeling
Source: Commun Biol. 2023 Jul 22;6:770. doi: 10.1038/s42003-023-05132-2 (PMC10363152; doi:10.1038/s42003-023-05132-2)
Supplement: Supplementary file 7 — Reporting Summary [file 42003_2023_5132_MOESM7_ESM.pdf]

nature portfolio

Corresponding author(s): Rajiv Chaturvedi

Last updated by author(s): Jun 21, 2023



# Reporting Summary

Nature Portfolio wishes to improve the reproducibility of the work that we publish. This form provides structure for consistency and transparency in reporting. For further information on Nature Portfolio policies, see our Editorial Policies and the Editorial Policy Checklist.

## Statistics

For all statistical analyses, confirm that the following items are present in the figure legend, table legend, main text, or Methods section.

| n/a | Confirmed | |
|---|---|---|
| ☐ | ☒ | The exact sample size (*n*) for each experimental group/condition, given as a discrete number and unit of measurement |
| ☐ | ☒ | A statement on whether measurements were taken from distinct samples or whether the same sample was measured repeatedly |
| ☐ | ☒ | The statistical test(s) used AND whether they are one- or two-sided<br>*Only common tests should be described solely by name; describe more complex techniques in the Methods section.* |
| ☐ | ☒ | A description of all covariates tested |
| ☐ | ☒ | A description of any assumptions or corrections, such as tests of normality and adjustment for multiple comparisons |
| ☐ | ☒ | A full description of the statistical parameters including central tendency (e.g. means) or other basic estimates (e.g. regression coefficient) AND variation (e.g. standard deviation) or associated estimates of uncertainty (e.g. confidence intervals) |
| ☐ | ☒ | For null hypothesis testing, the test statistic (e.g. *F*, *t*, *r*) with confidence intervals, effect sizes, degrees of freedom and *P* value noted<br>*Give P values as exact values whenever suitable.* |
| ☒ | ☐ | For Bayesian analysis, information on the choice of priors and Markov chain Monte Carlo settings |
| ☒ | ☐ | For hierarchical and complex designs, identification of the appropriate level for tests and full reporting of outcomes |
| ☒ | ☐ | Estimates of effect sizes (e.g. Cohen's *d*, Pearson's *r*), indicating how they were calculated |

*Our web collection on statistics for biologists contains articles on many of the points above.*

## Software and code

Policy information about availability of computer code

| | |
|---|---|
| Data collection | No software was used for data collection. |
| Data analysis | For differential expression analysis, bulk mRNA counts were normalized with RUV-seq (Risso et al. 2014). Differentially expressed miRNAs were identified using DESeq2 (v 1.30.1) (Love et al. 2014). Sequence similarity in other species was identified with “Single sequence search” function on miRBase (v22.1) with SSEARCH search method. Computationally predicted and experimentally validated target genes of differentially expressed miRNAs in AAo and LV were identified using multiMiR (v1.12.0) (Rue et al. 2014). Identified target genes were then converted from human to sheep orthologs using biomaRt (v2.46.3).<br>snRNA-seq reads were mapped using Cell Ranger v3 (10x Genomics). For a de novo annotation of the O. aries transcriptome, we followed the STAR alternate protocols 3 and 8 65, with the NCBI genome assembly 4.0 as a template (Dobin et al. 2015). The raw gene × nucleus read counts were normalized using SCTransform (Hafemeister et al. 2019). Seurat was used for snRNA-seq analysis (Butler et al. 2018). We used the Uniform Manifold Approximation and Projection (UMAP) for visualization (Becht et al. 2018). Multinomial logistic regression analyses were performed using scCODA (Buttner et al. 2021) and Dirichlet Reg (Maier et al. 2021). Pseudotime trajectories were measured using Slingshot (Street et al. 2018). We then used the tradeSeq package for trajectory analyses (Van den Berge et al. 2020). Pathway enrichment of these associated and differentially expressed genes was performed using gprofiler (Reimand et al. 2016). We performed cell-signaling analysis using the CellChat R package (Jin et al. 2021). |

For manuscripts utilizing custom algorithms or software that are central to the research but not yet described in published literature, software must be made available to editors and reviewers. We strongly encourage code deposition in a community repository (e.g. GitHub). See the Nature Portfolio guidelines for submitting code & software for further information.

## Data

Policy information about availability of data

All manuscripts must include a data availability statement. This statement should provide the following information, where applicable:

- Accession codes, unique identifiers, or web links for publicly available datasets
- A description of any restrictions on data availability
- For clinical datasets or third party data, please ensure that the statement adheres to our policy

Data availability: Sequence data is available through ArrayExpress (accession numbers: E-MTAB-12327 and E-MTAB-12230) and the Broad Institute (SCP1994). Additional data is available through https://doi.org/10.6084/m9.figshare.23511888.

## Research involving human participants, their data, or biological material

Policy information about studies with human participants or human data. See also policy information about sex, gender (identity/presentation), and sexual orientation and race, ethnicity and racism.

| | |
|---|---|
| Reporting on sex and gender | Due to the complex experiments (small number of biological replicates) and prenatal assessments, sex of the animals was not considered. |
| Reporting on race, ethnicity, or other socially relevant groupings | NA |
| Population characteristics | NA |
| Recruitment | Fetuses were chosen based on their lie in the uterus. |
| Ethics oversight | All procedures followed the Canadian Council on Animal Care guidelines and were approved by the University of Western Ontario Council on Animal Care (protocol 2010-257). |

Note that full information on the approval of the study protocol must also be provided in the manuscript.

# Field-specific reporting

Please select the one below that is the best fit for your research. If you are not sure, read the appropriate sections before making your selection.

☒ Life sciences ☐ Behavioural & social sciences ☐ Ecological, evolutionary & environmental sciences

For a reference copy of the document with all sections, see nature.com/documents/nr-reporting-summary-flat.pdf

# Life sciences study design

All studies must disclose on these points even when the disclosure is negative.

| | |
|---|---|
| Sample size | No sample size calculation was performed. |
| Data exclusions | One sample was excluded for cellular composition analyses as it was considered an outlier due to a high content of adipocytes (details in the methods section and Supplementary information). |
| Replication | We had four biological replicates of the coiled samples, and three biological replicates of the controls. |
| Randomization | Fetuses were randomly assigned to experimental groups. |
| Blinding | Blinding was not possible. |

# Reporting for specific materials, systems and methods

We require information from authors about some types of materials, experimental systems and methods used in many studies. Here, indicate whether each material, system or method listed is relevant to your study. If you are not sure if a list item applies to your research, read the appropriate section before selecting a response.

## Materials & experimental systems

| n/a | Involved in the study |
| --- | --- |
| ☒ | ☐ Antibodies |
| ☒ | ☐ Eukaryotic cell lines |
| ☒ | ☐ Palaeontology and archaeology |
| ☐ | ☒ Animals and other organisms |
| ☒ | ☐ Clinical data |
| ☒ | ☐ Dual use research of concern |
| ☒ | ☐ Plants |

## Methods

| n/a | Involved in the study |
| --- | --- |
| ☒ | ☐ ChIP-seq |
| ☒ | ☐ Flow cytometry |
| ☒ | ☐ MRI-based neuroimaging |

# Animals and other research organisms

Policy information about studies involving animals; ARRIVE guidelines recommended for reporting animal research, and Sex and Gender in Research

| | |
| --- | --- |
| Laboratory animals | Time-dated pregnant Dorset x Rideau Arcott ewes were studied (0.84 gestation). |
| Wild animals | Study did not involve wild animals. |
| Reporting on sex | Due to the complex experiments (small number of biological replicates) and prenatal assessments, sex of the animals was not considered. |
| Field-collected samples | Study did not involve field-collected samples. |
| Ethics oversight | All procedures followed the Canadian Council on Animal Care guidelines and were approved by the University of Western Ontario Council on Animal Care (protocol 2010-257). |

Note that full information on the approval of the study protocol must also be provided in the manuscript.
